# Critical requirement for BCR, BAFF, and BAFFR in memory B cell survival

**DOI:** 10.1084/jem.20191393

**Published:** 2020-10-29

**Authors:** Jennifer Müller-Winkler, Richard Mitter, Julie C.F. Rappe, Lesley Vanes, Edina Schweighoffer, Hamid Mohammadi, Andreas Wack, Victor L.J. Tybulewicz

**Affiliations:** 1 The Francis Crick Institute, London, UK; 2 Imperial College, London, UK

## Abstract

Memory B cells (MBCs) are long-lived cells that form a critical part of immunological memory, providing rapid antibody responses to recurring infections. However, very little is known about signals controlling MBC survival. Previous work has shown that antigen is not required for MBC survival, but a requirement for the B cell antigen receptor (BCR) has not been tested. Other studies have shown that, unlike naive B cells, MBCs do not express BAFFR and their survival is independent of BAFF, the ligand for BAFFR. Here, using inducible genetic ablation, we show that survival of MBCs is critically dependent on the BCR and on signaling through the associated CD79A protein. Unexpectedly, we found that MBCs express BAFFR and that their survival requires BAFF and BAFFR; hence, loss of BAFF or BAFFR impairs recall responses. Finally, we show that MBC survival requires IKK2, a kinase that transduces BAFFR signals. Thus, MBC survival is critically dependent on signaling from BCR and BAFFR.

## Teaser

Memory B cells are long-lived components of humoral immunological memory. Müller-Winkler et al. show that the survival of MBCs is critically dependent on signaling from the BCR, BAFF, BAFFR, and IKK2 kinase.

## Introduction

Immunological memory is characterized by the ability of the immune system to respond more rapidly and more robustly to a recurring infection. In the case of the humoral immune response, such a reexposure to a pathogen results in a secondary antibody response that, in comparison to a primary response, is quicker, larger in magnitude, and typified by higher-affinity antibodies. This humoral immunological memory arises from reservoirs of memory B cells (MBCs) and long-lived antibody-secreting plasma cells (PCs), which are established during a preceding primary immune response.

During a primary T-dependent antibody response, antigen-specific naive B cell clones selectively expand, supported by T cell help. Subsequently, these activated B cells and T helper cells migrate into the follicles of lymphoid organs where they establish germinal centers (GCs). In these structures, the B cells undergo somatic hypermutation of Ig variable regions, leading to an increase in affinity for antigen of the surface-bound Ig that makes up the B cell antigen receptor (BCR). Furthermore, GC B cells undergo class switch recombination, leading to a change from expressing the IgM and IgD forms of the BCR to other isotypes such as IgG1 (Shlomchik and Weisel, 2012; Suan et al., 2017). MBCs are generated from these activated B cells both before and after entry into the GC. Whereas substantial numbers of IgM^+^ MBCs are made before GC initiation, IgG1^+^ MBCs are preferentially generated in early GCs. In contrast, there is a continuous output of PCs during the late GC response (Inamine et al., 2005; Taylor et al., 2012; Weisel et al., 2016). In concordance with the timing of their egress from GCs, IgM^+^ and IgG1^+^ MBCs typically exhibit less somatically mutated Ig variable regions and therefore lower affinity for their cognate antigen than PCs (Kaji et al., 2012; Takahashi et al., 2001; Weisel et al., 2016). Whereas long-lived PCs reside in the bone marrow and maintain systemic levels of high-affinity antibodies (Nutt et al., 2015), MBCs represent a reservoir of quiescent cells bearing BCRs with low affinity for cognate antigen. MBCs are characterized by heterogeneity in both Ig mutation rates and expression levels of surface markers PD-L2, CD73, and CD80, which reflect variability in effector responses of MBCs following reactivation (Anderson et al., 2007; Tomayko et al., 2010). They can be reactivated by a broader selection of related and possibly mutated antigens and subsequently either undergo further affinity maturation in secondary GCs or rapidly secrete protective antibodies as short-lived plasmablasts (PBs; Dogan et al., 2009; Pape et al., 2011; Zuccarino-Catania et al., 2014).

To preserve humoral immunological memory, the antigen-specific reservoir of MBCs has to be maintained indefinitely. Studies have shown that MBCs are quiescent and long-lived, with many having a half-life in mice that is longer than the life span of the animal (Jones et al., 2015). Thus, pathways regulating MBC survival play a critical role in immunological memory; however, very little is known about them. Inhibition of the anti-apoptotic proteins Bcl-2, Bcl-X_L_, and Bcl-W revealed that IgG1^+^ MBC persistence is dependent on Bcl2-family proteins (Carrington et al., 2010; Torcia et al., 1996); however, external signals such as cytokines or receptors required for MBC longevity have not been identified (Weisel and Shlomchik, 2017).

Two crucial receptors mediating the survival of naive mature B cells are the BCR and BAFFR (TNFRSF13C; Schweighoffer and Tybulewicz, 2018). Several studies in naive B cells suggest that the BCR transduces a ligand-independent survival signal via its associated signaling components CD79A and CD79B and the tyrosine kinase SYK (Kraus et al., 2004; Lam et al., 1997; Schweighoffer et al., 2013). In contrast, it is not known if the BCR is required for the survival of MBCs. It has been suggested that trapping of cognate antigen via CD21/CD35 expressed on stromal cells and follicular (FO) dendritic cells is critical for the maintenance of MBCs, implying that BCR signals may be important for this process (Barrington et al., 2002). However, studies using either mice in which the BCR of MBC was changed to an irrelevant specificity or mice in which antigen could not be deposited in immune complexes on FO dendritic cells demonstrated that continued exposure to antigen is dispensable for MBC survival (Anderson et al., 2006; Hannum et al., 2000; Maruyama et al., 2000). Additionally, the presence of T cells is not required for MBC survival, implying that antigen recognition by T cells also does not contribute to MBC maintenance (Vieira and Rajewsky, 1990). However, studies have shown that SYK and phospholipase Cγ2 (PLCγ2), an enzyme that is phosphorylated and activated by SYK, are both required for MBC persistence (Ackermann et al., 2015; Hikida et al., 2009). Since both SYK and PLCγ2 transduce BCR signals, it is possible that the BCR is also required for MBC survival, potentially acting in an antigen-independent manner.

Binding of the cytokine BAFF (TNFSF13B) to BAFFR is crucial for the development and homeostasis of FO and marginal zone (MZ) B cells, implying that signaling from BAFFR is required for survival of these mature naive B cell subsets (Smulski and Eibel, 2018). The best characterized signaling pathway from BAFFR acts through TRAF2 and TRAF3 E3 ligases, resulting in the activation of the noncanonical NF-κB pathway via NF-κB–inducing kinase (NIK; MAP3K14) and IκB kinase 1 (IKK1). In addition, BAFFR signaling also activates the canonical NF-κB pathway via IKK2; however, the mechanism by which this happens is unknown (Rickert et al., 2011; Shinners et al., 2007). Whereas IKK1 and IKK2 are absolutely required for B cell maturation (Kaisho et al., 2001; Pasparakis et al., 2002), B cell survival is not affected by inducible deletion of IKK1 (Jellusova et al., 2013), and a requirement for IKK2 in the survival of mature B cells has not been tested. Interestingly, BAFFR has recently been shown to transduce survival signals partially via the BCR, SYK, and CD19 to the activation of the PI3K and ERK pathways; however, it is not known how this happens (Jellusova et al., 2013; Keppler et al., 2015; Schweighoffer et al., 2013).

Mouse MBCs have been reported to not express BAFFR (Benson et al., 2008). In agreement with this, blocking binding of BAFF to BAFFR in vivo resulted in a profound loss of naive B cells but did not affect MBC numbers (Benson et al., 2008). Another study reported mixed results in response to blocking BAFF, with one experiment showing a partial reduction in the numbers of IgM^+^ MBCs but no effect on numbers of IgG1^+^ MBCs, whereas other experiments showed no significant effect on IgM^+^ MBC or overall MBC numbers (Scholz et al., 2008). Transcriptional analysis of MBCs revealed that they express several receptors controlling stem cell renewal and quiescence, such as LIFR, BMPR1A, and ADORA2A, suggesting that BAFFR-mediated survival signals might be replaced by other, yet unknown, survival promoting factors (Luckey et al., 2006; Tomayko et al., 2008).

In this study, we investigate a potential role for the BCR and BAFFR in survival of IgM^+^ and IgG1^+^ MBCs and extend this to the analysis of proteins involved in transducing signals from these receptors. Importantly, we use an inducible genetic ablation system that allows us to first generate a population of antigen-specific MBCs and to then delete the gene encoding a protein of interest, thereby testing its role in MBC survival. We show that the BCR and the immune receptor tyrosine-based activation motif (ITAM) domain of its signaling subunit CD79A are both required for MBC survival in a cell-intrinsic manner. Surprisingly, we find that the persistence of MBCs is highly dependent on both BAFF and BAFFR and show that the requirement for BAFF extends to the survival of tissue-resident MBCs generated following viral infection. Finally, we show that the survival of IgM^+^ MBCs is partially dependent on IKK1, whereas survival of both IgM^+^ and IgG1^+^ MBCs is strongly dependent on NF-κB signaling via IKK2. Thus, we conclude that BCR, CD79A, BAFF, and BAFFR, as well as signaling via IKK2, are critical for MBC survival.

## Results

### Deletion of the BCR leads to loss of MBCs

**Figure S1.**
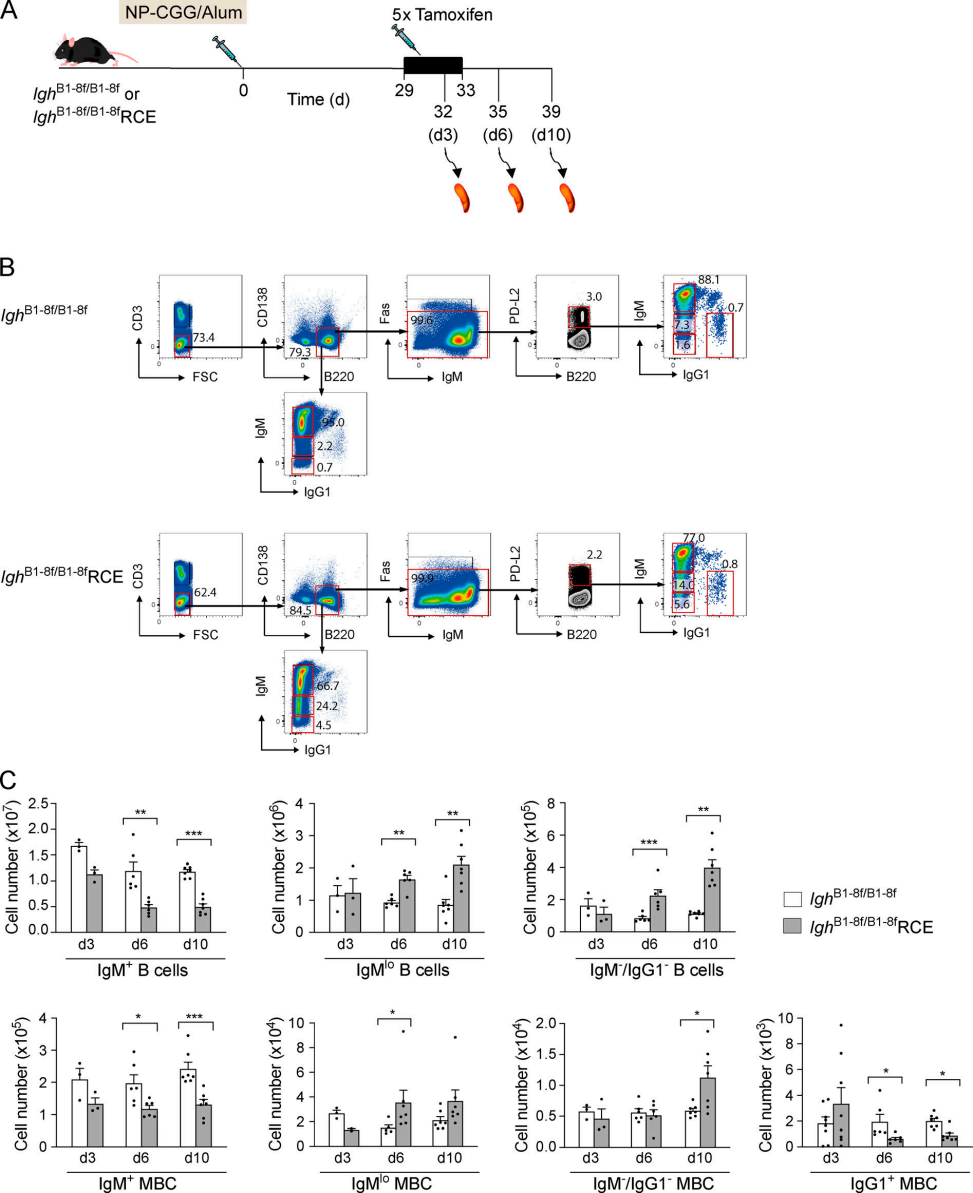
**Rapid loss of naive B cells and MBCs after tamoxifen administration.** **(A)** *Igh*^B1-8f/B1-8f^ or *Igh*^B1-8f/B1-8f^RCE mice were immunized with NP-CGG in alum and 29 d later injected with tamoxifen on five consecutive days. Spleens were analyzed 3, 6, and 10 d (d3, d6, and d10) after the first tamoxifen administration. **(B)** Flow cytometric analysis of splenocytes from mice immunized with NP-CGG in alum treated as described in A, showing gating strategy for IgM^+^, IgM^lo^, and IgM^−^/IgG1^−^ B cells (CD3^−^B220^+^CD138^−^) and IgM^+^, IgM^lo^, IgM^−^/IgG1^−^, and IgG1^+^ MBCs (CD3^−^B220^+^CD138^−^Fas^−^PD-L2^+^) 10 d after the first tamoxifen administration. Numbers in dot plots indicate percentages of cell populations within gates (red boxes). **(C)** Mean (±SEM) numbers of total IgM^+^, IgM^lo^, and IgM^−^/IgG1^−^ B cells and IgM^+^, IgM^lo^, IgM^−^/IgG1^−^, and IgG1^+^ MBCs in spleens of mice immunized with NP-CGG in alum and analyzed at 3, 6, and 10 d (d3, *n* = 3; d6, *n* = 6; and d10, *n* = 7) after the first tamoxifen administration. Each dot represents one mouse. Data from two independent experiments are pooled. Mann-Whitney test was used for statistical analysis. *, 0.01 < P < 0.05; **, 0.001 < P < 0.01; ***, 0.0001 < P < 0.001.

To investigate if the BCR is required for MBC survival, we used mice with the *Igh*^B1-8f^ allele, in which a rearranged VDJ gene segment flanked by loxP sites (floxed) has been inserted into the Ig heavy chain (IgH) locus (Lam et al., 1997). This encodes a heavy chain protein that has binding specificity for 4-hydroxy-3-nitrophenyl acetyl (NP), and in the resulting mouse ∼5% of B cells express an NP-specific (NP^+^) BCR. To allow inducible deletion of the VDJ gene, we crossed *Igh*^B1-8f/+^ mice to ROSA26-CreER^T2^ (RCE) mice that express a tamoxifen-inducible Cre recombinase (de Luca et al., 2005), thereby generating *Igh*^B1-8f/B1-8f^RCE mice in which tamoxifen treatment should result in rapid loss of the BCR from the surface of all B cells, including MBCs. To test this, we immunized mice with NP–chicken γ-globulin (NP-CGG) in alum to generate MBCs and, starting 29 d later, injected the mice five times daily with tamoxifen. Within 10 d of the start of tamoxifen injection, there was an ∼50% reduction in the number of B cells expressing IgM and MBCs expressing IgM or IgG1, consistent with the loss of IgH expression (Fig. S1, A–C). This was accompanied by the appearance of IgM^lo^ B cells and MBCs, accounting for around 15–20% of the starting numbers of these cell types and a small population of IgM^−^IgG1^−^ B cells accounting for a further 5% of cell numbers. Thus, deletion of the BCR results in reduced numbers of MBCs, consistent with a requirement for the BCR for MBC survival.

**Figure 1.**
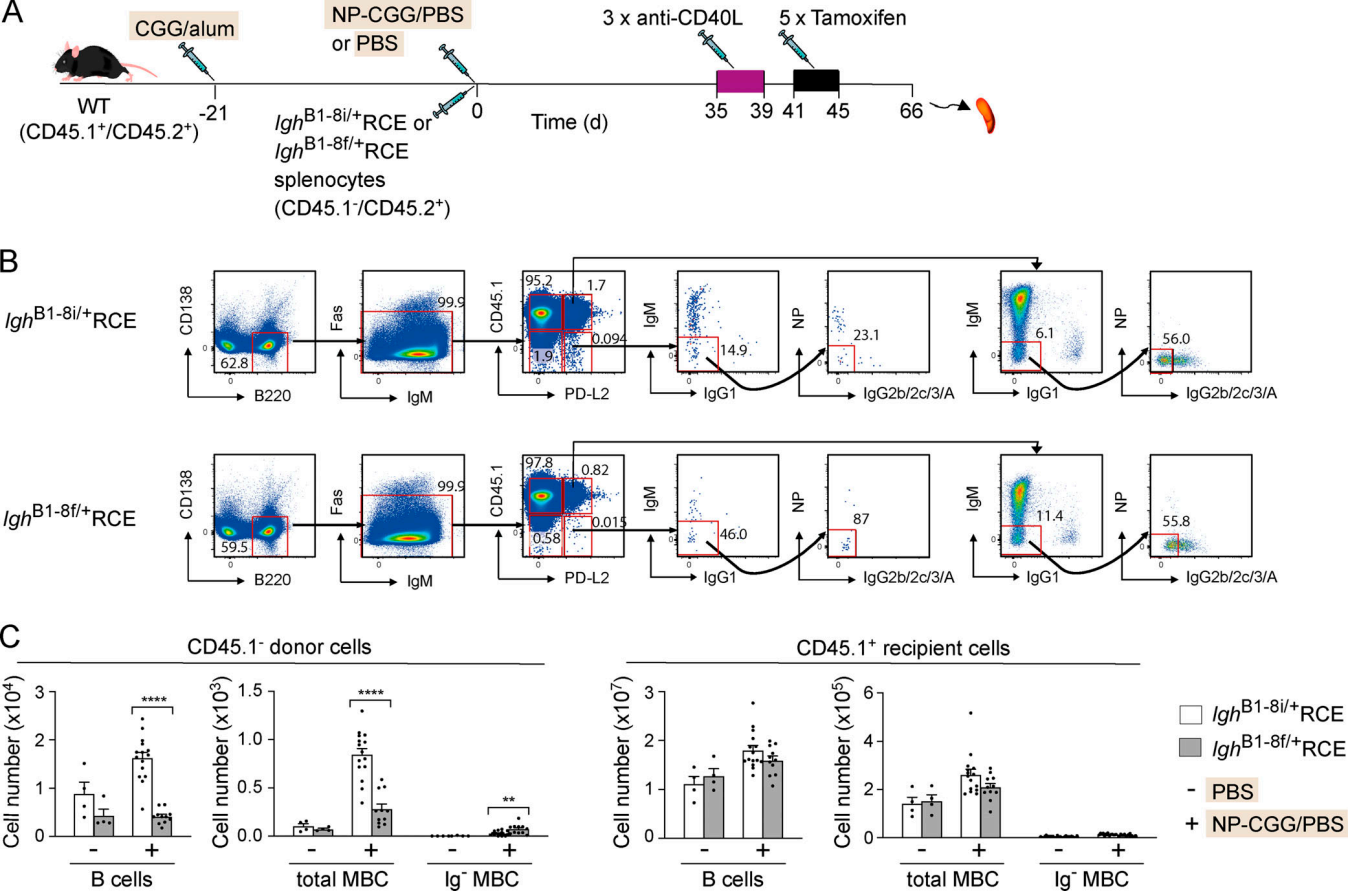
**Deletion of the BCR leads to loss of MBCs.** **(A)** WT (CD45.1^+^CD45.2^+^) mice were immunized with CGG in alum. 21 d later (on day 0), splenocytes from *Igh*^B1-8i/+^RCE or *Igh*^B1-8f/+^RCE donor mice (CD45.1^−^CD45.2^+^) were transferred into recipient mice that were also immunized on the same day with NP-CGG in PBS or PBS alone. Starting on day 35, mice were injected on 3 alternate d with anti-CD40L, followed by five daily tamoxifen injections, and spleens were analyzed on day 66. **(B)** Flow cytometric analysis of splenocytes from mice treated as described in A, showing gating strategy for total naive B cells (B220^+^CD138^−^Fas^−^PD-L2^−^), total MBCs (B220^+^CD138^−^Fas^−^PD-L2^+^), and Ig^−^ MBCs (B220^+^CD138^−^Fas^−^PD-L2^+^IgM^−^IgG1^−^IgG2b/2c/3/A^−^NP^−^). Expression of CD45.1 was used to separate donor (CD45.1^−^) and recipient (CD45.1^+^) cells. Numbers in dot plots indicate percentages of cell populations within gates (red boxes). **(C)** Mean (±SEM) numbers of donor and recipient total naive B cells and of total and Ig^−^ MBCs in spleens of mice immunized with NP-CGG (+; *n* = 15 for* Igh*^B1-8i/+^RCE and *n* = 11 for *Igh*^B1-8f/+^RCE) or PBS alone (−; *n* = 4). Each dot represents one mouse. Data pooled from two independent experiments. Mann-Whitney test was used for statistical analysis. **, 0.001 < P < 0.01; ****, P < 0.0001.

In view of the large reduction in the numbers of naive B cells following deletion of the BCR, it is possible that any observed change in the numbers of MBCs could be secondary to the extensive death of naive B cells and the resulting perturbation of lymphoid tissue architecture and not to cell-intrinsic loss of the BCR. To address this, we turned to an adoptive transfer approach. We made use of *Igh*^B1-8f/+^RCE B cells, almost all of which express the floxed VDJ gene because of allelic exclusion, and as a control for Cre activity, we used *Igh*^B1-8i/+^RCE B cells; the *Igh*^B1-8i^ allele has the same NP^+^ rearranged VDJ gene segment inserted into the IgH locus as *Igh*^B1-8f^, but without loxP sites (Sonoda et al., 1997). We transferred *Igh*^B1-8f/+^RCE or *Igh*^B1-8i/+^RCE splenocytes into congenically marked WT mice, which 21 d earlier had been immunized with CGG in alum to provide T cell help for the incoming NP^+^ B cells, and then immunized them with NP-CGG (Fig. 1 A). This immunization protocol gives a robust T-dependent antibody response, resulting in the production of both IgM^+^ and switched IgG1^+^ NP^+^ MBCs. 35 d after immunization, the mice were treated with an anti-CD40L antibody to block CD40–CD40L interactions between B and T cells, thereby stopping T cell help and GC reactions. This ensured that no new MBCs could be made; thus, all NP^+^ MBCs analyzed after this time were generated during the first 4 wk after immunization (Ackermann et al., 2015). Finally, the mice were treated with tamoxifen to induce deletion of the floxed VDJ region and thus abrogate expression of the BCR, and they were analyzed 3 wk later, 66 d after immunization with NP-CGG. As expected, the total number of donor *Igh*^B1-8f/+^RCE B cells decreased substantially compared with *Igh*^B1-8i/+^RCE B cells (Fig. 1, B and C). Furthermore, in mice that had been immunized with NP-CGG, the number of donor MBCs also decreased substantially (Fig. 1, B and C).

We used expression of PD-L2 to identify MBCs since it is not expressed on naive B cells and 80–90% of IgG1^+^ MBCs express this marker (Zuccarino-Catania et al., 2014). We noted that some Ig-expressing MBCs remained after tamoxifen treatment, indicating incomplete deletion of the floxed VDJ gene segment by the tamoxifen-inducible Cre. Nevertheless, since there had been a loss of the majority of Ig-expressing MBCs, if the BCR was not required for MBC survival, we would expect a substantial rise in the number of MBCs that lacked surface Ig (Ig^−^). However, we saw no such rise, indicating that BCR deletion led to loss of MBCs (Fig. 1 C). In contrast, there was no change in the numbers of recipient B cells or MBCs, demonstrating a cell-intrinsic requirement for the BCR in maintaining MBC numbers.

### BCR-mediated signaling is required for the maintenance of MBCs

**Figure 2.**
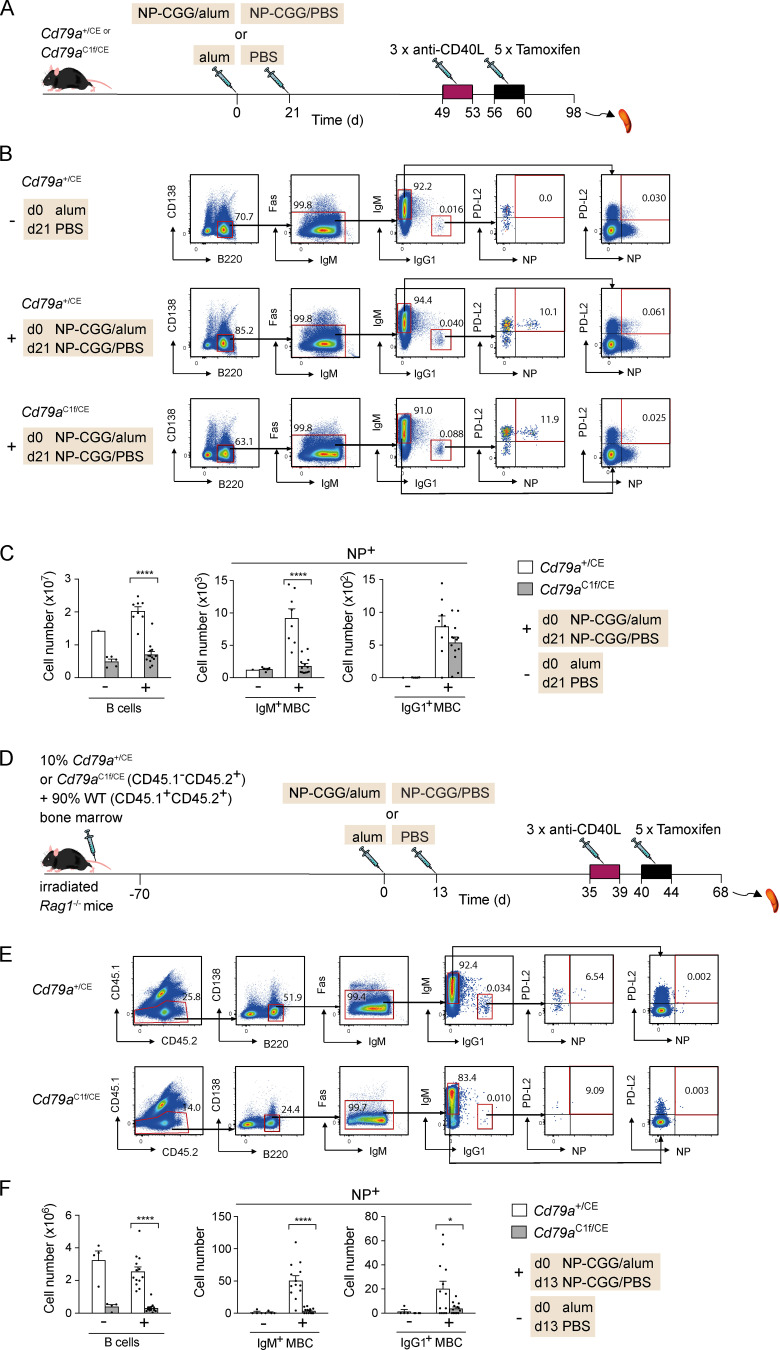
**Loss of BCR-mediated signaling leads to reduction of MBCs.** **(A)** *Cd79a^+^*^/CE^ or *Cd79a*^C1f/CE^ mice were immunized with NP-CGG in alum or alum alone on day 0 and rechallenged with NP-CGG in PBS or PBS alone 21 d later. Mice were injected with anti-CD40L on days 49, 51, and 53, followed by five daily tamoxifen injections. **(B)** Flow cytometric analysis of splenocytes from mice immunized with NP-CGG in alum or alum alone, treated as described in A, showing gating strategy for total B cells (B220^+^CD138^−^) and NP^+^ IgM^+^ and IgG1^+^ MBCs (B220^+^CD138^−^Fas^−^PD-L2^+^). Numbers in dot plots indicate percentages of cell populations within gates (red boxes). **(C)** Mean (±SEM) numbers of total B cells and NP^+^ IgM^+^ and IgG1^+^ MBCs in spleens of mice immunized with NP-CGG in alum (+; *n* = 8 for *Cd79a^+^*^/CE^ and *n* = 13 for *Cd79a*^C1f/CE^) or alum alone (−; *n* = 1 for *Cd79a^+^*^/CE^ and *n* = 5 for *Cd79a*^C1f/CE^). Each dot represents one mouse. **(D)** Mixed bone marrow chimeras were generated by reconstituting irradiated *Rag1*^−/−^ mice with 10% bone marrow from *Cd79a*^+/CE^ or *Cd79a*^C1f/CE^ (CD45.1^−^CD45.2^+^) and 90% WT bone marrow (CD45.1^+^CD45.2^+^) for 70 d. Mice were immunized with NP-CGG in alum or alum alone on day 0 and rechallenged with NP-CGG in PBS or PBS alone 13 d later. Mice were injected with anti-CD40L on days 35, 37, and 39, followed by five daily tamoxifen injections. **(E and F)** Flow cytometric analysis of total B cells and MBC populations from mice treated as described in D and immunized with NP-CGG in alum (+; *n* = 13) or alum alone (−; *n* = 4). Analysis as in B and C, except that cells were also gated for CD45.1^−^CD45.2^+^ cells. Data pooled from two independent experiments (C and F). Mann-Whitney test was used for statistical analysis. *, 0.01 < P < 0.05; ****, P < 0.0001.

The previous experiment did not distinguish whether signaling through the BCR is essential to maintain MBC numbers or if the BCR has a different nonsignaling function. To address this directly, we used *Cd79a*^C1f^ mice, in which exon 4 of the *Cd79a* gene is floxed (Kraus et al., 2004). CD79A and CD79B are transmembrane proteins associated with the BCR that are critical for signaling from the receptor (Kurosaki, 2002). Exon 4 of *Cd79a* codes for the ITAM domain of CD79A, a region of the cytoplasmic domain of the protein that is essential for transducing BCR signals. We crossed mice with the *Cd79a*^C1f^ allele to mice in which a tamoxifen-inducible CreER^T2^ had been inserted into the *Cd79a* gene (mb1-CreER^T2^, *Cd79a*^CE^; Hobeika et al., 2015) to generate *Cd79a*^C1f/CE^ mice in which tamoxifen treatment results in deletion of the ITAM domain of CD79A but leaves the rest of the protein intact. Importantly, this means the BCR can still reach the cell surface. *Cd79a*^C1f/CE^ and *Cd79a*^+/CE^ mice were immunized and boosted with NP-CGG to generate a pool of MBCs, then treated with anti-CD40L to block GCs, and then with tamoxifen to delete the CD79A ITAM (Fig. 2 A). Analysis showed that mutation of CD79A resulted in a substantial reduction in the number of total B cells and of NP^+^ IgM^+^ MBCs in *Cd79a*^C1f/CE^ mice compared with *Cd79a*^+/CE^ controls, while the reduction in NP^+^ IgG1^+^ MBC numbers was not significant (Fig. 2, B and C).

**Figure S2.**
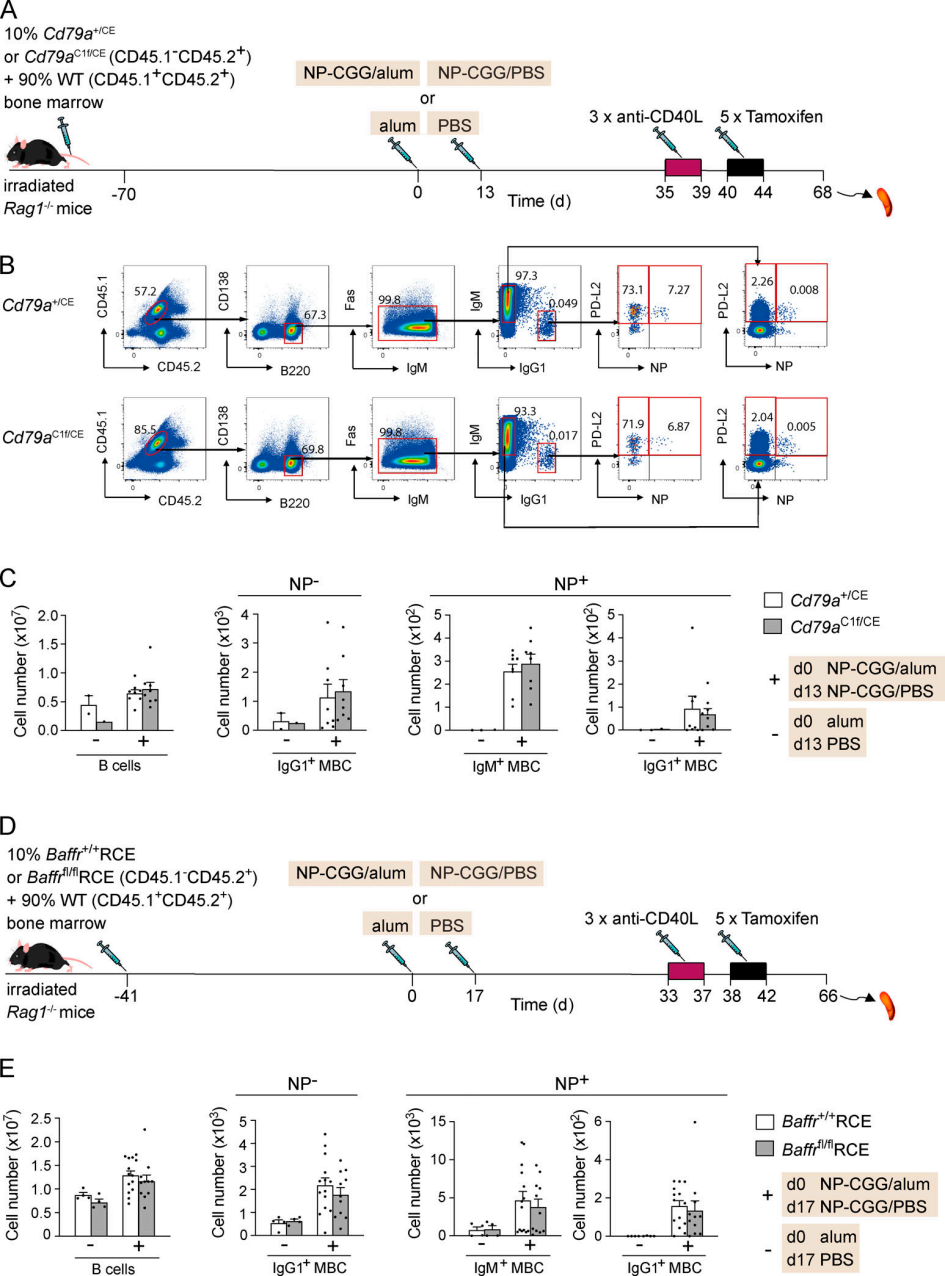
**No loss of WT B cells or MBCs in bone marrow chimeras containing a mixture of WT and CD79A mutant B cells or WT and BAFFR-deficient B cells.** **(A)****Mixed bone marrow chimeras were generated by reconstituting irradiated *Rag1*^−/−^ mice with 10% bone marrow from *Cd79a*^+/CE^ or *Cd79a*^C1f/CE^ (CD45.1^−^CD45.2^+^) and 90% WT bone marrow (CD45.1^+^CD45.2^+^) for 70 d. Mice were immunized with NP-CGG in alum or alum alone on day 0 and rechallenged with NP-CGG in PBS or PBS alone 13 d later. Mice were injected with anti-CD40L on days 35, 37, and 39, followed by five daily tamoxifen injections. **(B)** Flow cytometric analysis of splenocytes from mice immunized with NP-CGG in alum treated as described in A, showing gating strategy for the WT (CD45.1^+^CD45.2^+^) total B cells (B220^+^CD138^−^), NP^−^ IgG1^+^ MBCs, and NP^+^ IgM^+^ and IgG1^+^ MBCs (B220^+^CD138^−^Fas^−^PD-L2^+^). Numbers in dot plots indicate percentages of cell populations within gates (red boxes). **(C)** Mean (±SEM) numbers of WT (CD45.1^+^CD45.2^+^) total B cells, NP^−^ IgG1^+^ MBCs, and NP^+^ IgM^+^ and IgG1^+^ MBCs in spleens of mice immunized with NP-CGG in alum (+; *n* = 8) or alum alone (−; *n* = 2 for *Cd79a*^+/CE^ and *n* = 1 for *Cd79a*^C1f/CE^). **(D)** Mixed bone marrow chimeras were generated by reconstituting irradiated *Rag1*^−/−^ mice with 10% bone marrow from *Baffr*^+/+^RCE or *Baffr*^fl/fl^RCE (CD45.1^−^CD45.2^+^) and 90% WT bone marrow (CD45.1^+^CD45.2^+^) for 41 d. Mice were immunized with NP-CGG in alum or alum alone on day 0 and boosted with NP-CGG in PBS or PBS alone 17 d later. Mice were injected with anti-CD40L on days 33, 35, and 37, followed by five daily tamoxifen injections. **(E)** Mean (±SEM) numbers of WT (CD45.1^+^CD45.2^+^) total B cells (B220^+^CD138^−^), NP^−^ IgG1^+^ MBCs, and NP^+^ IgM^+^ and IgG1^+^ MBCs (B220^+^CD138^−^Fas^−^PD-L2^+^) in spleens of mice immunized with NP-CGG in alum (+; *n* = 14 for *Baffr*^+/+^RCE and *n* = 11 for *Baffr*^fl/fl^RCE) or alum alone (−; *n* = 4), treated as described in D. The gating strategy is the same as in B. Each dot represents one mouse; data are from one of two independent experiments (C) or pooled from two independent experiments (E). Mann-Whitney test was used for statistical analysis. There were no statistically significant differences in numbers of WT B cells or MBCs between the chimeras reconstituted with 10% *Cd79a*^+/CE^ compared with 10% *Cd79a*^C1f/CE^ bone marrow or 10% *Baffr*^+/+^RCE compared with 10% *Baffr*^fl/fl^RCE bone marrow.

To establish if the requirement for the CD79A ITAM domain is cell intrinsic, we generated mixed bone marrow chimeras by reconstituting RAG1-deficient mice with a mixture of bone marrow from either *Cd79a*^+/CE^ or *Cd79a*^C1f/CE^ mice (10%) and from congenically marked WT mice (90%; Fig. 2 D). As before, these chimeras were immunized and boosted with NP-CGG to generate MBCs and treated with anti-CD40L and tamoxifen. Tamoxifen treatment resulted in a very large decrease in *Cd79a*^C1f/CE^ B cells and NP^+^ IgM^+^ and IgG1^+^ MBCs compared with *Cd79a*^+/CE^ MBCs (Fig. 2, E and F). In contrast, the number of WT B cells and MBCs in the same mixed chimeras was unaffected (Fig. S2, A–C). Thus, IgM^+^ and IgG1^+^ MBCs have a cell-intrinsic requirement for BCR signaling through CD79A for their maintenance.

### MBCs express BAFFR and TACI but not B cell maturation antigen (BCMA)

**Figure S3.**
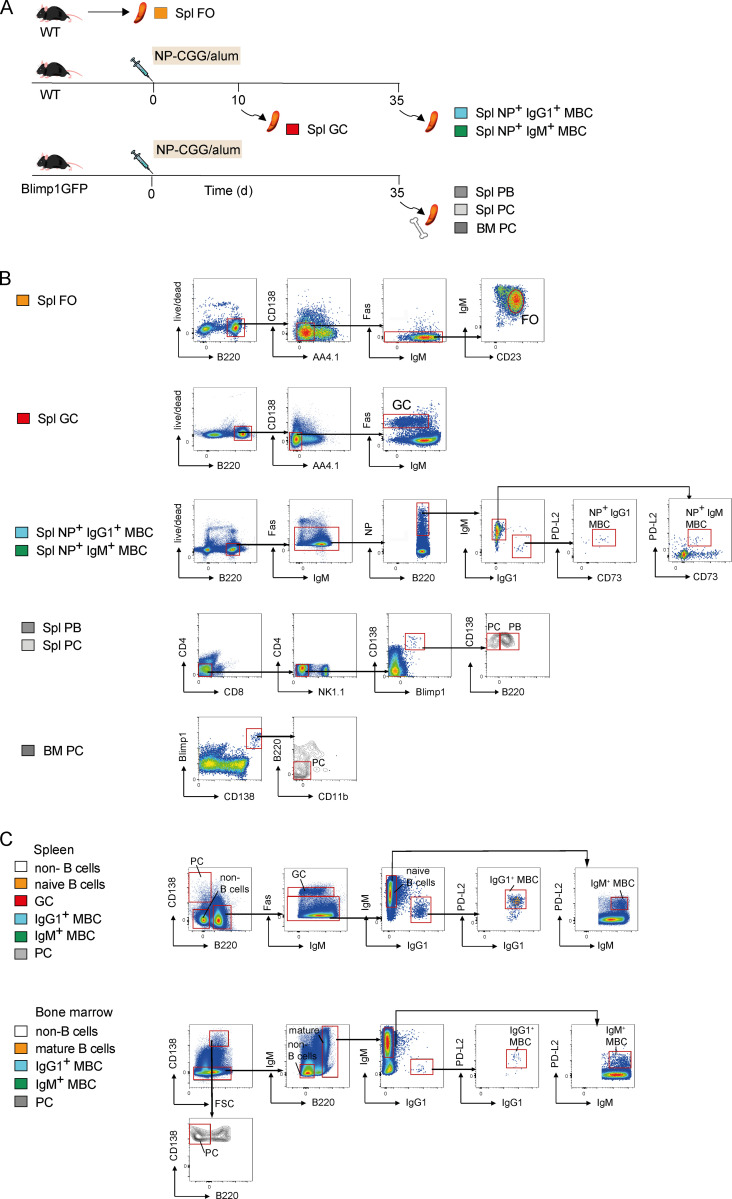
**Flow cytometric gating strategy for B cell populations sorted for RNAseq analysis and for analysis of BAFFR and TACI expression.** **(A)** Spleens were harvested from WT mice or from WT or Blimp1GFP mice that were immunized with NP-CGG in alum on day 0, followed by dissection of spleen or bone marrow on days 10 and 35. **(B)** FO B cells (Spl FO, B220^+^CD138^−^AA4.1^−^Fas^−^IgM^lo^CD23^+^) from spleens of unimmunized WT mice, GC B cells (Spl GC; B220^+^CD138^−^AA4.1^−^Fas^+^) from WT spleens 10 d after immunization with NP-CGG in alum, NP^+^ IgM^+^ MBCs (Spl NP^+^ IgM^+^ MBC; B220^+^Fas^−^NP^+^CD73^+^PD-L2^+^IgM^+^IgG1^−^), and IgG1^+^ MBCs (Spl NP^+^ IgG1^+^ MBC; B220^+^Fas^−^NP^+^CD73^+^PD-L2^+^IgM^−^IgG1^+^) from WT spleens 35 d after immunization with NP-CGG in alum and splenic PBs (Spl PB, CD4^−^CD8^−^NK1.1^−^CD138^hi^Blimp1^hi^B220^+^), splenic PCs (Spl PC, CD4^−^CD8^−^NK1.1^−^CD138^hi^Blimp1^hi^B220^−^), and bone marrow PCs (BM PC, CD138^hi^Blimp1^hi^B220^−^CD11b^−^) from Blimp1GFP mice 35 d after immunization with NP-CGG in alum were flow sorted and analyzed by RNAseq. Three to five mice were pooled to obtain the required cell numbers for each biological replicate of each population. Red boxes indicate gates used to isolate subsets of cells. **(C)** Flow cytometric analysis of splenocytes and bone marrow from WT mice immunized with NP-CGG in alum as described in A for the analysis of BAFFR and TACI expression shown in Fig. 3, B and C, showing gating strategy for total splenic (B220^−^CD138^−^) and bone marrow (B220^−^CD138^−^IgM^−^) non–B cells, splenic (PC; B220^−^CD138^hi^) and bone marrow PCs (FCShiB220^−^CD138^hi^), splenic GC B cells (GC; CD138^−^B220^+^Fas^+^), splenic naive B cells (B220^+^CD138^−^Fas^−^IgM^+^), bone marrow mature B cells (B220^hi^CD138^−^), splenic IgM^+^ and IgG1^+^ MBCs (B220^+^CD138^−^Fas^−^PD-L2^+^), and bone marrow IgM^+^ and IgG1^+^ MBCs (B220^hi^CD138^−^PD-L2^+^). Red boxes indicate gates used to isolate subsets of cells.

**Figure 3.**
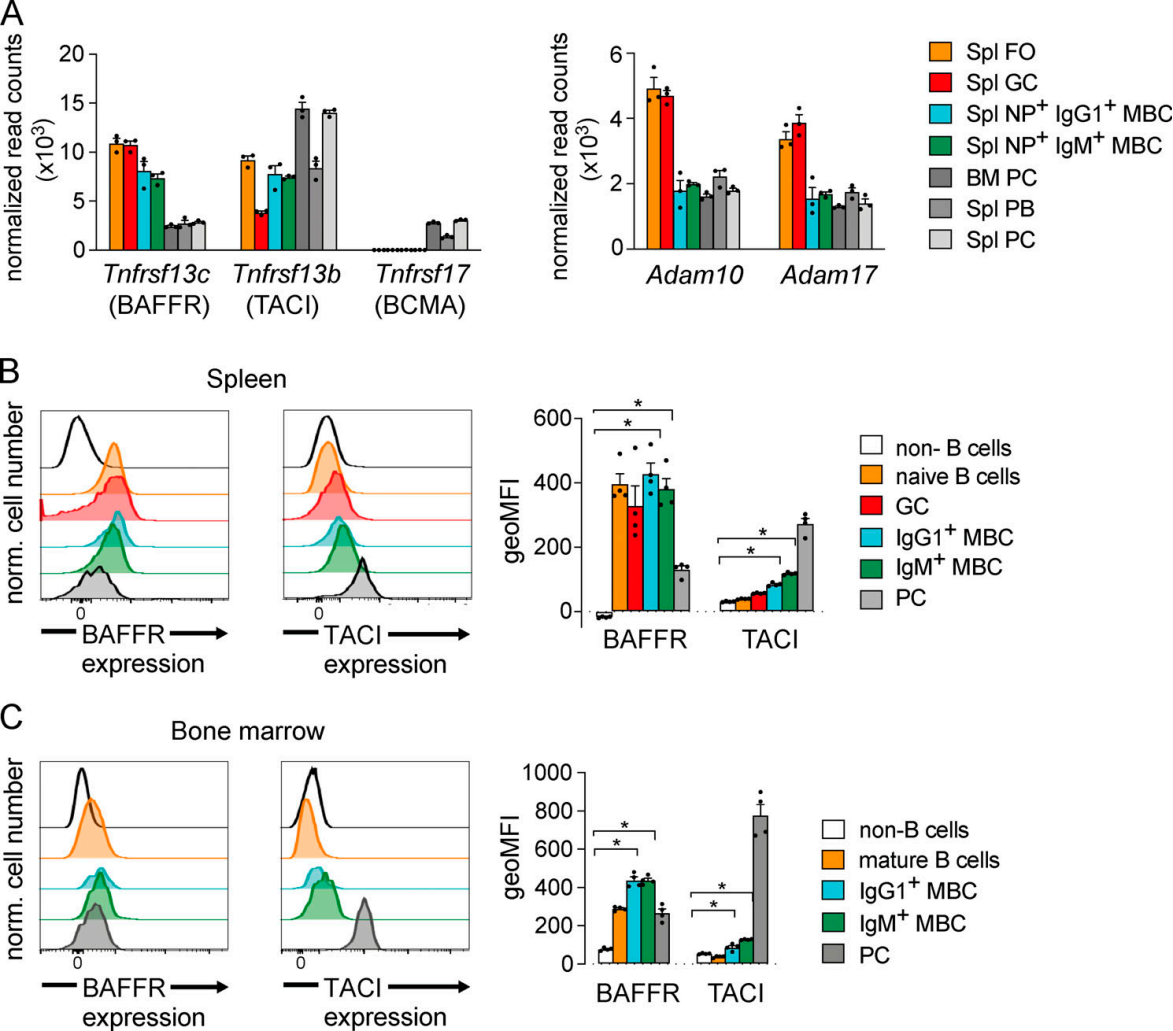
**MBCs express BAFFR and TACI, but not BCMA.** **(A)** Mean (±SEM) normalized read counts as a measure of expression of mRNA for *Tnfrsf13c* (BAFFR), *Tnfrsf13b* (TACI), *Tnfrsf17* (BCMA), *Adam10*, and *Adam17* determined by RNAseq in the following B cell populations: splenic FO B cells (Spl FO, B220^+^CD138^−^AA4.1^−^IgM^lo^CD23^+^) from WT mice, splenic GC B cells (Spl GC, B220^+^CD138^−^AA4.1^−^Fas^+^) from mice 10 d after immunization with NP-CGG in alum, NP^+^ IgM^+,^ and IgG1^+^ MBCs (Spl NP^+^ IgM^+^ or IgG1^+^ MBC; B220^+^Fas^−^NP^+^CD73^+^PD-L2^+^) from mice 35 d after immunization with NP-CGG in alum, and splenic PBs (Spl PBs, CD4^−^CD8^−^NK1.1^−^CD138^hi^Blimp1^hi^B220^+^), splenic PCs (Spl PCs, CD4^−^CD8^−^NK1.1^−^CD138^hi^Blimp1^hi^B220^−^), and bone marrow PCs (BM PCs, CD138^hi^Blimp1^hi^B220^−^CD11b^−^) from Blimp1GFP mice 35 d after immunization with NP-CGG in alum (see Fig. S3, A and B for gating); *n* = 3 mice for each population. Each dot represents one biological replicate consisting of three to five pooled mice. **(B and C)** Flow cytometric analysis of BAFFR and TACI surface expression on splenic (B) and bone marrow (C) non–B cells (CD138^−^B220^−^), naive or mature B cells (CD138^−^B220^+^IgM^+^), GC B cells (CD138^−^B220^+^Fas^+^), NP^+^ IgM^+^ or IgG1^+^ MBCs (CD138^−^B220^+^Fas^−^NP^+^PD-L2^+^), and PCs (CD138^hi^B220^−^) 35 d after immunization of WT mice with NP-CGG in alum (*n* = 4; see Fig. S3 C for gating). Bar graphs show the mean (±SEM) of the geometric mean fluorescence intensity (geoMFI) of BAFFR and TACI surface levels. Each dot represents one mouse; data shown from one of three independent experiments. Mann-Whitney test was used for statistical analysis. *, 0.01 < P < 0.05. norm., normalized.

In addition to BCR signaling, the survival of naive mature B cells depends on signaling from BAFFR induced by binding of BAFF (Rickert et al., 2011). Previous reports had suggested that MBCs do not express BAFFR and do not depend on BAFF or BAFFR for their survival (Benson et al., 2008; Scholz et al., 2008). To explore this issue further, we investigated the expression of the genes for the BAFF-binding receptors BAFFR (*Tnfrsf13c*), TACI (*Tnfrsf13b*), and BCMA (*Tnfrsf17*) in MBCs using RNA sequencing (RNAseq) and compared this to the expression in naive splenic FO B cells, GC B cells, PBs, and PCs and in bone marrow PC (Fig. S3, A and B; and Table S1). We found that IgM^+^ and IgG1^+^ MBCs express similar levels of both *Tnfrsf13c* and *Tnfrsf13b* as FO and GC B cells and express no *Tnfrsf17* (Fig. 3 A). In contrast, PBs and PCs express lower levels of *Tnfrsf13c* but have substantial expression of *Tnfrsf13b* and *Tnfrsf17*. We also looked at expression of *Adam10* and *Adam17*, which code for metalloproteases that cleave BAFFR from the cell surface following ligand binding, thereby reducing the duration of responses to BAFF (Smulski et al., 2017). Interestingly, MBCs express lower amounts of both *Adam10* and *Adam17* than do FO B cells, which may make them more responsive to BAFF (Fig. 3 A).

We extended this analysis using flow cytometry to measure surface levels of BAFFR and TACI. We found that splenic IgM^+^ and IgG1^+^ MBCs express similar amounts of BAFFR as naive splenic B cells (Fig. 3 B). In the bone marrow, which has been shown to harbor MBCs in humans (Giesecke et al., 2014; Paramithiotis and Cooper, 1997), BAFFR expression is slightly higher on IgM^+^ and IgG1^+^ MBCs than on mature B cells (Fig. 3 C). TACI expression is significantly higher on MBCs than on mature B cells in both spleen and bone marrow but does not reach the level found on PCs (Fig. 3, B and C). Together, these findings suggest that both IgM^+^ and IgG1^+^ MBCs express BAFFR and TACI but not BCMA and that they express at least as much BAFFR as naive mature B cells.

### BAFFR is required for the maintenance of MBCs

**Figure 4.**
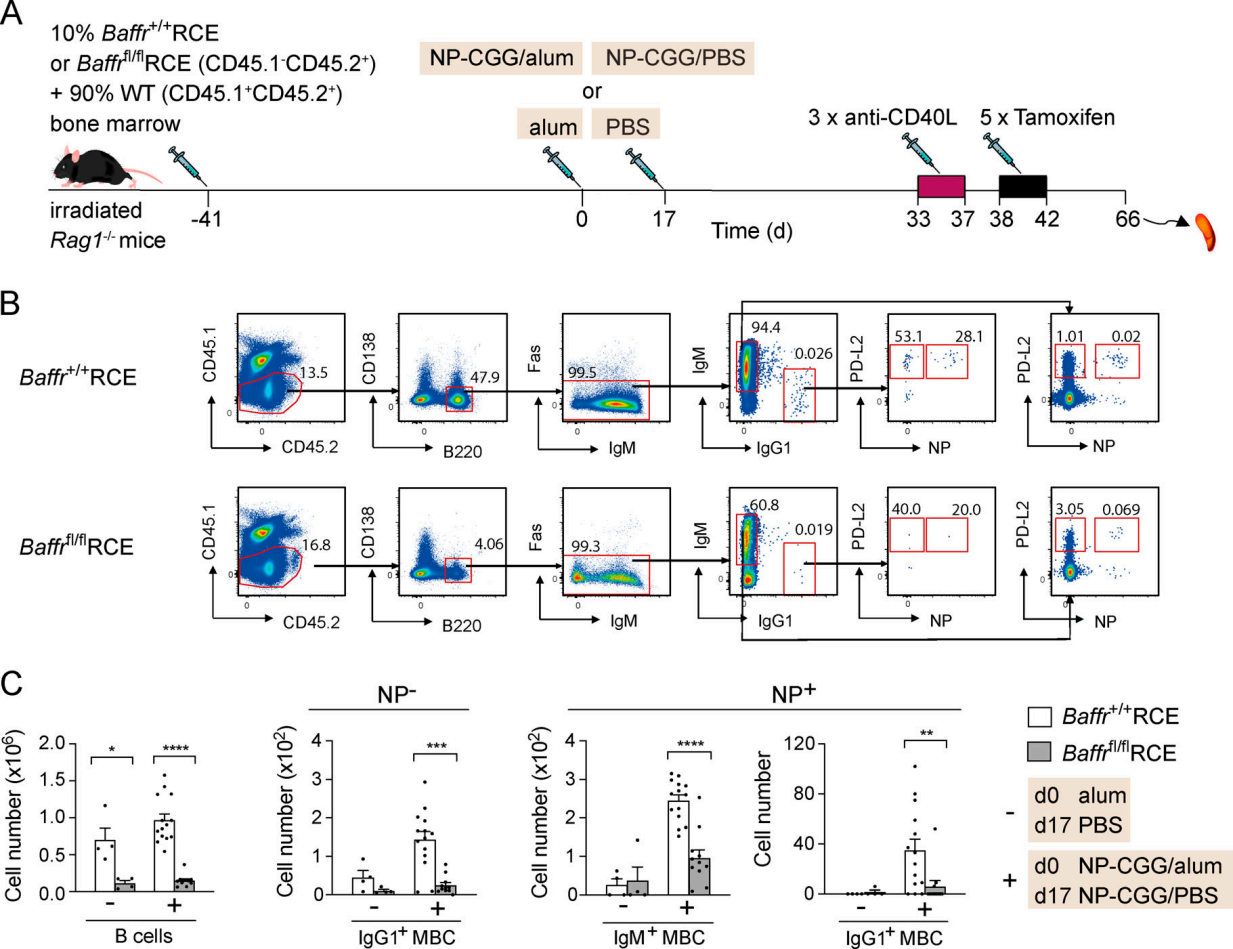
**BAFFR deletion results in the loss of MBCs.** **(A)** Mixed bone marrow chimeras were generated by reconstituting irradiated *Rag1*^−/−^ mice with 10% bone marrow from *Baffr*^+/+^RCE or *Baffr*^fl/fl^RCE (CD45.1^−^CD45.2^+^) and 90% WT bone marrow (CD45.1^+^CD45.2^+^) for 41 d. Mice were immunized with NP-CGG in alum or alum alone on day 0 and boosted with NP-CGG in PBS or PBS alone 17 d later. Mice were injected with anti-CD40L on days 33, 35, and 37, followed by five daily tamoxifen injections. **(B)** Flow cytometric analysis of splenocytes from mice immunized with NP-CGG in alum treated as described in A and analyzed on day 66, showing gating strategy for CD45.1^−^CD45.2^+^ total naive B cells (B220^+^CD138^−^Fas^−^PD-L2^−^) and for NP^−^ IgG1^+^ MBCs and NP^+^ IgM^+^ and IgG1^+^ MBCs (B220^+^CD138^−^Fas^−^PD-L2^+^). Numbers in dot plots indicate percentages of cell populations within gates (red boxes). **(C)** Mean (±SEM) numbers of total naive B cells and of NP^−^ IgG1^+^ and NP^+^ IgM^+^ and IgG1^+^ MBCs in spleens of mice immunized with NP-CGG in alum (+; *n* = 14 for *Baffr*^+/+^RCE and *n* = 11 for *Baffr*^fl/fl^RCE) or alum alone (−; *n* = 4). Each dot represents one mouse. Data pooled from two independent experiments. Mann-Whitney test was used for statistical analysis. *, 0.01 < P < 0.05; **, 0.001 < P < 0.01; ***, 0.0001 < P < 0.001; ****, P < 0.0001.

Given the expression of BAFFR on MBCs, we decided to test its requirement for MBC maintenance using a genetic ablation strategy. We made use of *Baffr*^fl/fl^ mice in which exons 3 and 4 of the *Tnfrsf13c* gene are floxed (Sasaki et al., 2004) and crossed these to mice carrying the RCE allele, which expresses a tamoxifen-inducible Cre recombinase. Deletion of these exons results in a frameshift and hence loss of BAFFR expression. To investigate a cell-intrinsic requirement for BAFFR in MBC survival, we generated mixed bone marrow chimeras by reconstituting RAG1-deficient mice with a mixture of bone marrow from either *Baffr*^+/+^RCE or *Baffr*^fl/^^fl^RCE mice (10%) and from congenically marked WT mice (90%; Fig. 4 A). After immunization with NP-CGG in alum or with alum alone and a subsequent boost with NP-CGG or PBS, *Baffr*^+/+^RCE or *Baffr*^fl/fl^RCE chimeric mice were treated with anti-CD40L and then with tamoxifen to delete BAFFR. Analysis showed that loss of BAFFR on *Baffr*^fl/fl^RCE cells resulted in a strong reduction in the number of mature B cells and of NP^+^ IgM^+^ and IgG1^+^ MBCs and NP-nonspecific (NP^−^) IgG1^+^ MBCs compared with control *Baffr*^+/+^RCE cells (Fig. 4, B and C). In contrast, WT B cells and MBCs in the same mixed chimeras showed no significant changes in MBC numbers (Fig. S2, D and E). Thus, both IgM^+^ and IgG1^+^ MBCs require BAFFR for their survival, and this requirement is cell intrinsic.

### BAFF depletion results in the loss of MBCs

**Figure 5.**
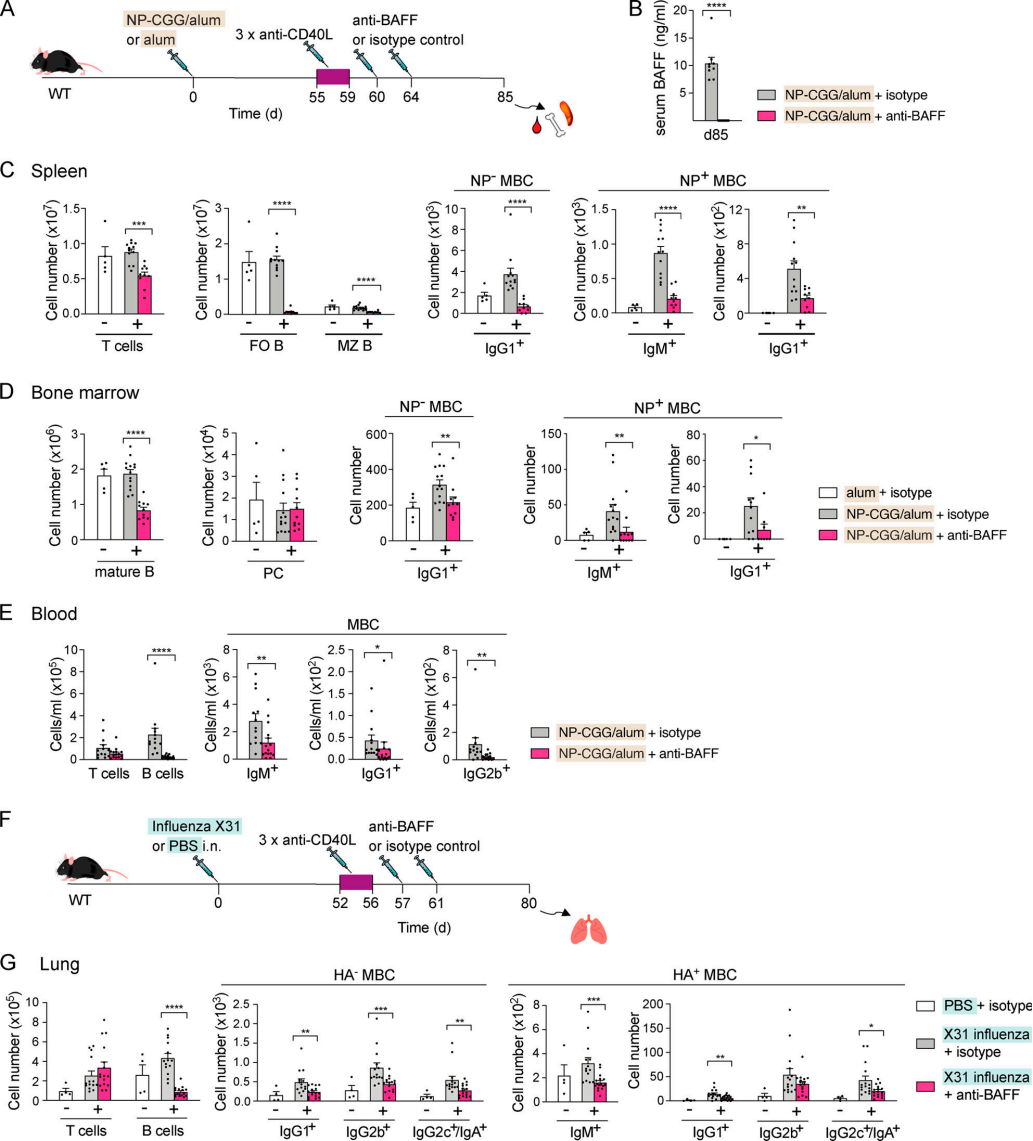
**BAFF depletion results in the loss of MBCs.** **(A)** WT mice were immunized with NP-CGG in alum or alum alone on day 0, followed by anti-CD40L or isotype control antibody injections on days 55, 57, and 59. Anti-BAFF or isotype control antibody was injected on days 60 and 64. **(B)** Mean (±SEM) BAFF levels in serum of mice immunized with NP-CGG in alum 21 d after anti-BAFF (*n* = 11) or isotype control antibody (*n* = 9) injection. Each dot represents one mouse. **(C–E)** Mean (±SEM) numbers of B cell populations in spleen (C), bone marrow in two legs (D), or blood (E) of mice immunized with NP-CGG in alum (+; *n* = 10 for anti-BAFF and *n* = 12 for isotype control antibody in spleen; *n* = 11 for anti-BAFF and *n* = 14 for isotype control antibody in bone marrow; and *n* = 15 for anti-BAFF and *n* = 13 for isotype control antibody in blood) or alum alone (−; *n* = 5) as described in A, 21 d after last anti-BAFF or isotype control antibody injection. Splenic T cells (CD3^+^CD138^−^B220^−^), FO (FO B, CD3^−^CD138^−^B220^+^Fas^−^AA4.1^−^PD-L2^−^IgM^lo^CD23^+^), MZ (MZ B, CD3^−^CD138^−^B220^+^Fas^−^AA4.1^−^PD-L2^−^IgM^hi^CD23^−^), and NP^+^ IgG1^+^ and IgM^+^ MBCs and NP^−^ IgG1^+^ MBCs (CD3^−^CD138^−^B220^+^Fas^−^AA4.1^−^PD-L2^+^), bone marrow mature B cells (CD138^−^B220^hi^IgM^+^), PCs (B220^−^CD138^hi^), and NP^−^ IgG1^+^ MBCs and NP^+^ IgM^+^ and IgG1^+^ MBCs (CD138^−^B220^hi^PD-L2^+^), and blood T cells (CD3^+^B220^−^), B cells (CD3^−^B220^+^CD138^−^), and IgM^+^, IgG1^+^, or IgG2b^+^ MBCs (CD3^−^B220^+^CD138^−^PD-L2^+^) were analyzed using the gating strategy shown in Fig. S4 B. Each dot represents one mouse. **(F)** WT mice were infected i.n. with X31 influenza or given PBS as a control on day 0, followed by anti-CD40L or isotype control antibody injections on days 52, 54, and 56. Anti-BAFF or isotype control antibody was injected on days 57 and 61. **(G)** Mean (±SEM) numbers of cell populations in lungs of mice infected with X31 influenza (+; *n* = 16 for anti-BAFF and *n* = 14 for isotype control antibody) or given PBS as a control (−; *n* = 4) 19 d after last anti-BAFF or isotype control antibody injection. Pulmonary T cells (CD3^+^CD19^−^), total B cells (CD3^−^CD138^−^CD19^+^), and HA^+^ IgM^+^, IgG1^+^, IgG2b^+^, and IgG2c^+^/IgA^+^ and HA^−^ IgG1^+^, IgG2b^+^, and IgG2c^+^/IgA^+^ MBCs (CD3^−^CD138^−^CD19^+^PD-L2^+^) were analyzed using the gating strategy shown in Fig. S4 D. Data pooled from two (C–E) and three (G) independent experiments. Mann-Whitney test was used for statistical analysis. *, 0.01 < P < 0.05; **, 0.001 < P < 0.01; ***, 0.0001 < P < 0.001; ****, P < 0.0001.

**Figure S4.**
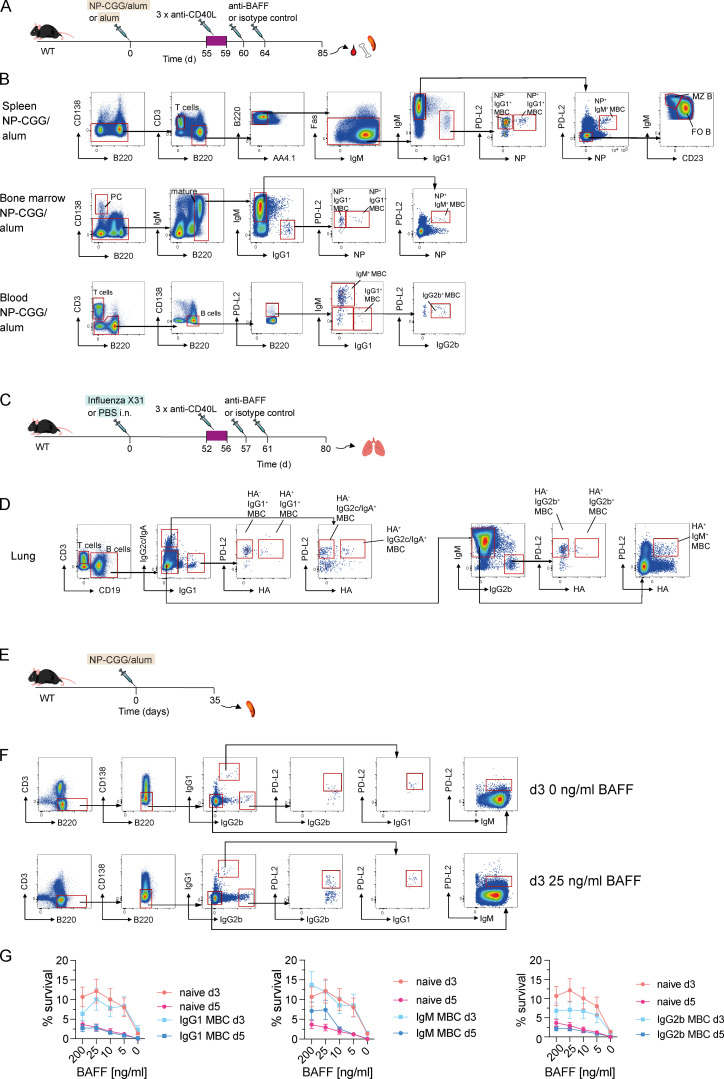
**Flow cytometric gating strategy for B cell populations in spleen and bone marrow of BAFF-depleted mice and BAFF-dependent survival of MBCs in vitro.** **(A)** WT mice were immunized with NP-CGG in alum or alum alone on day 0, followed by anti-CD40L or isotype control antibody injections on days 55, 57, and 59. Anti-BAFF or isotype control antibody was injected on days 60 and 64. **(B)** Gating strategy for splenic T cells (CD138^−^CD3^+^B220^−^), FO (FO B, CD3^−^CD138^−^B220^+^Fas^−^AA4.1^−^PD-L2^−^IgM^lo^IgG1^−^CD23^+^NP^−^), MZ (MZ B, CD3^−^CD138^−^B220^+^Fas^−^AA4.1^−^PD-L2^−^IgM^hi^IgG1^−^CD23^−^NP^−^), NP^+^ IgM^+^ MBCs (IgM^+^ MBC, CD3^−^CD138^−^B220^+^Fas^−^AA4.1^−^PD-L2^+^IgM^+^IgG1^−^), NP^−^ and NP^+^ IgG1^+^ MBCs (IgG1^+^ MBC, CD3^−^CD138^−^B220^+^Fas^−^AA4.1^−^PD-L2^+^IgM^−^IgG1^+^), bone marrow mature B cells (CD138^−^B220^hi^), PCs (CD138^hi^B220^−^), NP^+^ IgM^+^ MBCs (CD138^−^B220^hi^PD-L2^+^IgM^+^IgG1^−^), NP^−^, and NP^+^ IgG1^+^ MBCs (CD138^−^B220^hi^PD-L2^+^IgM^−^IgG1^+^), blood T cells (CD3^+^B220^−^), total B cells (CD3^−^CD138^−^B220^+^), and IgM^+^, IgG1^+^, and IgG2b^+^ MBCs (CD3^−^CD138^−^B220^+^PD-L2^+^) in mice immunized with NP-CGG in alum treated as described in A 21 d after last injection of isotype control antibody. **(C)** WT mice were infected i.n. with X31 influenza or given PBS as a control on day 0, followed by anti-CD40L or isotype control antibody injections on days 52, 54, and 56. Anti-BAFF or isotype control antibody was injected on days 57 and 61. **(D)** Gating strategy for pulmonary T cells (CD3^+^CD19^−^), total B cells (CD3^−^CD19^+^), HA^+^ IgM^+^ MBCs, and HA^+^ and HA^−^ IgG1^+^, IgG2b^+^, and IgG2c^+^/IgA^+^ MBCs (CD3^−^CD138^−^CD19^+^PD-L2^+^) in lungs of mice infected with X31 influenza and then injected twice with isotype control antibody as described in C and analyzed 19 d after the last injection. **(E)** WT mice were immunized with NP-CGG in alum, and spleens were harvested 35 d later. **(F)** Flow cytometric analysis of WT splenocytes, immunized with NP-CGG in alum as described in E and cultured in 0 ng/ml or 25 ng/ml BAFF for 3 d (d3), showing gating strategy for total B cells (CD3^−^CD138^−^B220^+^), IgG1^+^, IgG2b^+^, and IgM^+^ MBCs (CD3^−^CD138^−^B220^+^PD-L2^+^). **(G)** Mean (±SEM) percent survival of total B cells and IgG1^+^, IgG2b^+^, and IgM^+^ MBCs at days 3 (d3; *n* = 12) and 5 (d5; *n* = 12) normalized to total cell numbers at day 0, cultured in BAFF at the indicated concentrations. Data pooled from three independent experiments (G). Mann-Whitney test was used for statistical analysis. Red boxes indicate gates used to identify cell populations.

Given the unexpected finding that BAFFR is required for MBC maintenance, we investigated if BAFF was also essential for MBC homeostasis. We immunized WT mice with NP-CGG in alum or with alum alone, treated them with anti-CD40L 55 d later, and then injected anti-BAFF or an isotype control antibody 60 and 64 d after immunization and analyzed the animals 21 d later (Fig. 5 A). BAFF levels in the serum of mice injected with anti-BAFF were greatly reduced 21 d after injection compared with animals treated with isotype control antibody, confirming the efficient depletion of BAFF (Fig. 5 B). In the spleen, anti-BAFF treatment resulted in a small change in T cell numbers, whereas the numbers of FO and MZ B cells, as well as NP^+^ IgM^+^ and IgG1^+^ MBCs and NP^−^ IgG1^+^ MBCs were strongly decreased (Fig. 5 C and Fig. S4, A and B). In the bone marrow, anti-BAFF treatment resulted in no change in PC numbers but a substantial reduction in the numbers of mature B cells and IgM^+^ and IgG1^+^ MBCs (Fig. 5 D and Fig. S4 B). Injection of humans with an anti-BAFF antibody results in a transient increase in MBCs in the blood, suggesting that MBCs were relocating from lymphoid tissues into the circulation (Stohl et al., 2012). In contrast, we found significantly reduced numbers of naive B cells and IgM^+^, IgG1^+^, and IgG2b^+^ MBCs in the blood of mice treated with anti-BAFF (Fig. 5 E and Fig. S4 B). Thus, BAFF is required for the maintenance of MBCs in the spleen, the bone marrow, and the circulation.

### BAFF is required for the maintenance of lung-resident MBCs following influenza infection

To investigate if the dependence of MBCs on BAFF extends to tissue-resident MBCs generated in response to a physiological stimulus, we infected mice i.n. with the X31 influenza virus or gave them PBS as a control, treated them with anti-CD40L 52 d later, and then injected anti-BAFF or an isotype control antibody 57 and 61 d after infection (Fig. 5 F). Analysis of the mice 19 d later showed that BAFF depletion had no impact on the number of pulmonary T cells, whereas the numbers of lung-resident B cells as well as hemagglutinin-specific (HA^+^) IgM^+^ MBCs and HA^+^ and nonspecific (HA^−^) MBCs expressing IgG1, IgG2b, IgG2c, or IgA isotypes were significantly reduced (Fig. 5 G and Fig. S4, C and D). The reduction of HA^+^ and HA^−^ MBCs was also observed in the spleens of X31 influenza–infected mice after BAFF depletion (data not shown). Thus, lung-resident MBCs generated in response to influenza infection require BAFF for their maintenance.

### Similar BAFF-dependent survival of MBCs and naive B cells in vitro

Many MBCs have a half-life that is much longer than that of naive B cells (Jones et al., 2015), implying that they must differ in their survival mechanisms. While we have shown that MBCs, like naive B cells, require BAFF and BAFFR for survival, it is possible that MBCs respond much better to BAFF, thereby explaining their improved survival in the animal. To test this, we cultured MBCs and naive B cells in the presence of a range of BAFF concentrations and measured cell survival after 3 or 5 d. We found no significant difference in BAFF-dependent survival of MBCs compared with naive B cells, indicating that altered responsiveness to BAFF does not account for the differences in life span of these two cell types, at least as assessed using an in vitro survival assay (Fig. S4, F and G).

### Depletion of BAFF impairs antibody recall responses

**Figure 6.**
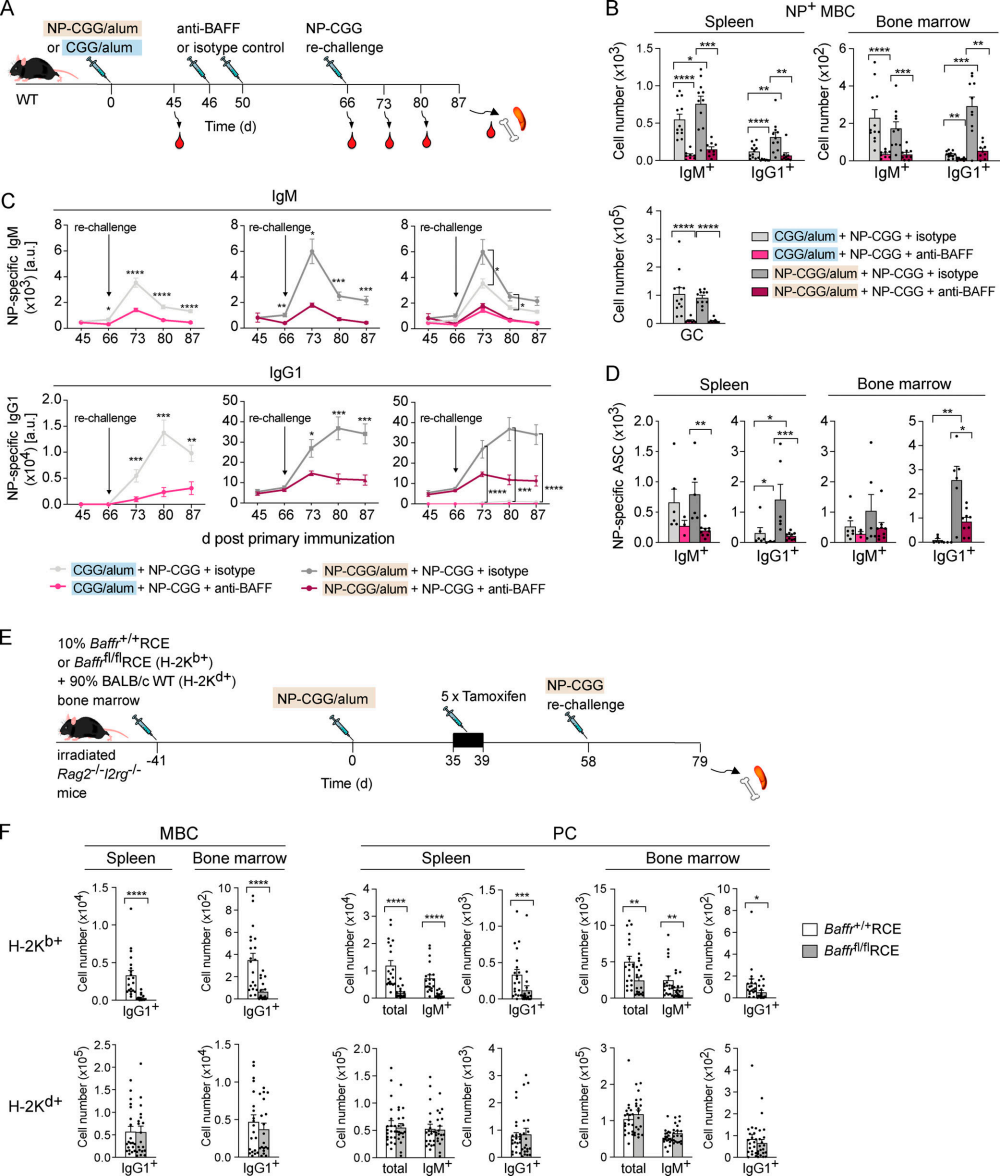
**BAFF depletion results in impaired recall responses due to loss of MBCs.** **(A)** WT mice were immunized with CGG or NP-CGG in alum on day 0, followed by anti-BAFF or isotype control antibody injections 46 d and 50 d later and rechallenged with NP-CGG 16 d later. Blood was withdrawn 45, 66, 73, 80, and 87 d after primary immunization, and spleen and bone marrow were analyzed at day 87. **(B)** Mean (±SEM) numbers of NP^+^ IgM^+^ and IgG1^+^ MBCs in spleen and bone marrow and GC B cells in the spleen of mice immunized with CGG in alum (*n* = 8 for anti-BAFF and *n* = 12 for isotype control antibody in spleen and bone marrow) or NP-CGG in alum (*n* = 9 for anti-BAFF and *n* = 11 for isotype control antibody in spleen; *n* = 9 for anti-BAFF and *n* = 10 for isotype control antibody in bone marrow) as described in A, 21 d after rechallenge (gating as in Fig. S4 B). **(C)** Mean (±SEM) concentration of NP^+^ IgM and IgG1 in serum of mice immunized with CGG in alum (*n* = 8 for anti-BAFF and *n* = 12 for isotype control antibody) or NP-CGG in alum (*n* = 9 for anti-BAFF and *n* = 10 for isotype control antibody) treated as described in A. For clarity, the responses of mice primed with CGG or NP-CGG are shown separately as well as together. a.u., arbitrary unit. **(D)** Mean (±SEM) numbers of NP^+^ IgM^+^ and IgG1^+^ antibody-secreting cells (ASCs) in spleen (*n* = 3 for anti-BAFF and *n* = 6 for isotype control antibody for mice immunized with CGG in alum; *n* = 9 for anti-BAFF and *n* = 6 for isotype control antibody for mice immunized with NP-CGG in alum) and bone marrow (*n* = 3 for anti-BAFF and *n* = 7 for isotype control antibody for mice immunized with CGG in alum; *n* = 9 for anti-BAFF and *n* = 6 for isotype control antibody for mice immunized with NP-CGG in alum) of mice treated as described in A, 21 d after rechallenge with NP-CGG, determined by ELISPOT (Fig. S5 B). **(E)** Mixed bone marrow chimeras were generated by reconstituting irradiated *Rag2*^−/−^*Il2rg*^−/−^ mice with 10% bone marrow from *Baffr*^+/+^RCE or *Baffr*^fl/fl^RCE B6 mice (H-2K^b+^) and 90% bone marrow from WT BALB/c mice (H-2K^d+^) for 41 d. Mice were immunized with NP-CGG in alum on day 0, given five daily tamoxifen injections starting on day 35, and rechallenged with NP-CGG in PBS at day 58. Spleen and bone marrow were taken for analysis on day 79. **(F)** Mean (±SEM) numbers of H-2K^b+^ or H-2K^d+^ MBCs and PCs (*n* = 20 for *Baffr*^+/+^RCE and *n* = 19 for *Baffr*^fl/fl^RCE; gating as in Fig. S5 D). Data pooled from two (B–D) and three (F) independent experiments. Mann-Whitney test was used for statistical analysis. *, 0.01 < P < 0.05; **, 0.001 < P < 0.01; ***, 0.0001 < P < 0.001; ****, P < 0.0001.

**Figure S5.**
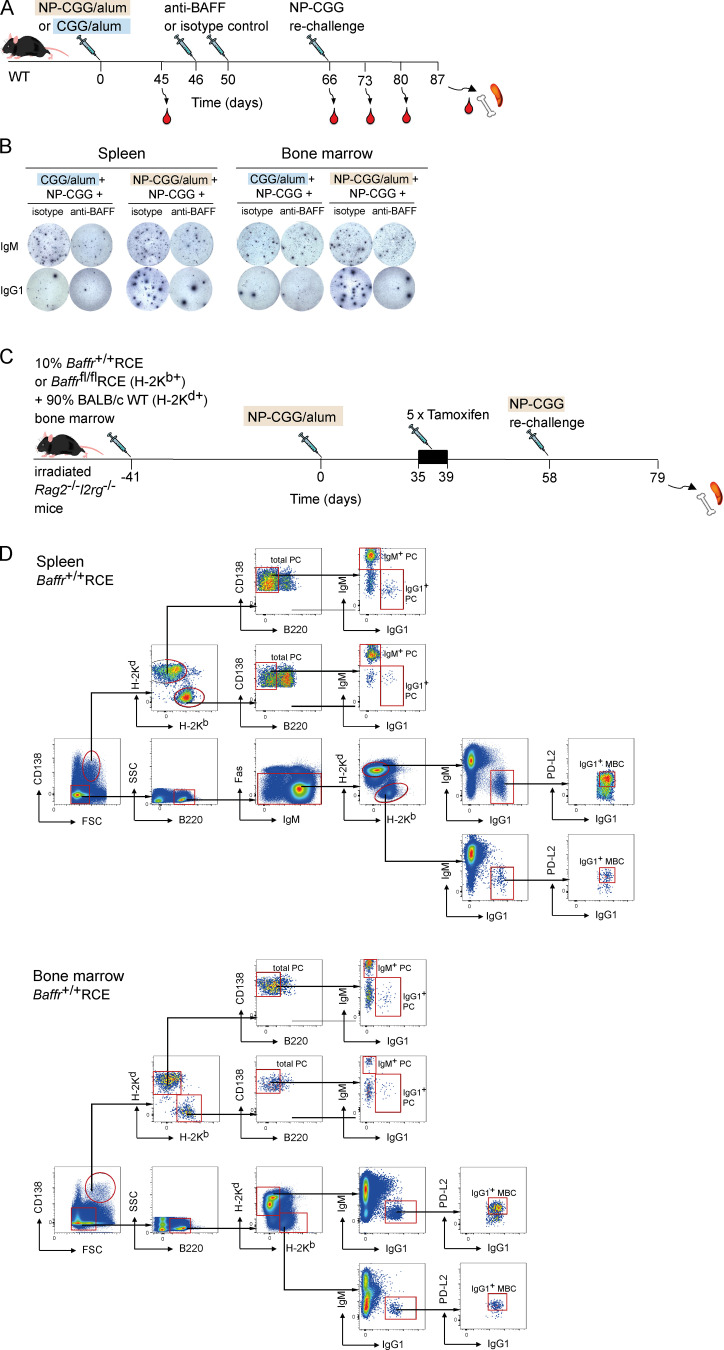
**Analysis of recall responses in mice treated with anti-BAFF or following deletion of BAFFR.** **(A)** WT mice were immunized with CGG or NP-CGG in alum on day 0, followed by anti-BAFF or isotype control antibody injections 46 d and 50 d later and rechallenged with NP-CGG 16 d later. Blood was withdrawn 45, 66, 73, 80, and 87 d after primary immunization, and spleen and bone marrow were analyzed at day 87. **(B)** Images of example ELISPOT wells showing NP^+^ IgM^+^ or IgG1^+^ antibody secreting cells in spleen and bone marrow from mice treated as indicated. Each spot corresponds to a single antibody secreting cell. **(C)** Mixed bone marrow chimeras were generated by reconstituting irradiated *Rag2*^−/−^*Il2rg*^−/−^ mice with 10% bone marrow from *Baffr*^+/+^RCE or *Baffr*^fl/fl^RCE B6 mice (H-2K^b+^) and 90% bone marrow from WT BALB/c mice (H-2K^d+^) for 41 d. Mice were immunized with NP-CGG in alum on day 0, given five daily tamoxifen injections starting on day 35, and rechallenged with NP-CGG in PBS on day 58. Spleen and bone marrow were taken for analysis on day 79. **(D)** Flow cytometric analysis showing gating of H-2K^b+^ or H-2K^d+^ splenic IgG1^+^ MBCs (CD138^−^B220^+^Fas^−^PD-L2^+^) and bone marrow IgG1^+^ MBCs (CD138^−^B220^+^PD-L2^+^) as well as splenic and bone marrow IgM^+^ and IgG1^+^ PCs (FSC^hi^CD138^hi^B220^−^). Red boxes indicate gates used to identify cell populations. FSC, forward scatter; SSC, side scatter.

A hallmark of immunological memory is a faster and larger response to a secondary immune challenge. To examine if the loss of MBCs in BAFF-depleted mice has an impact on this recall response, we immunized WT mice with NP-CGG in alum, treated them with anti-BAFF or isotype control antibody 46 d later, and rechallenged them with NP-CGG a further 20 d later (Fig. 6 A). We collected blood from the mice before the anti-BAFF injection and then before and after rechallenge and analyzed the spleen and bone marrow 21 d after rechallenge. Since the antibody response after rechallenge could be derived either from reactivation of MBCs or from de novo activation of naive B cells, we also generated control mice in which the primary immunization was with CGG in alum; thus, any NP^+^ B cell response after NP-CGG rechallenge would be generated from naive B cells. Analysis at the end of the protocol (day 87) showed that anti-BAFF treatment greatly reduced the numbers of NP^+^ IgM^+^ and IgG1^+^ MBCs in the spleen and bone marrow and splenic GC cells after rechallenge (Fig. 6 B). Comparison of CGG- and NP-CGG–immunized mice showed that most of the IgM^+^ MBCs were derived from naive B cells activated during rechallenge, whereas most of the IgG1^+^ MBCs were derived from reactivated MBCs. Furthermore, anti-BAFF treatment resulted in a very large drop in both NP^+^ IgM and IgG1 in the serum and in the numbers of NP^+^ IgM- and IgG1-secreting cells in the spleen and the bone marrow (Fig. 6, C and D; and Fig. S5, A and B). Once again, most of the IgG1 response was due to reactivation of MBCs, whereas a substantial fraction of the IgM response resulted from de novo activation of naive B cells. Thus, BAFF is required for the generation of a robust antigen-specific antibody recall response from MBCs.

To exclude that the impaired antibody recall response could be secondary to the large loss of B cells and altered lymphoid architecture induced by loss of BAFF, rather than to a direct cell-intrinsic effect on MBCs and their reactivation, we generated mixed bone marrow chimeras by reconstituting mice deficient in RAG2 and γ_c_ with a mixture of bone marrow from either *Baffr*^+/+^RCE or *Baffr*^fl/fl^RCE B6 mice (10%, H-2K^b+^) and from haplotypically distinct BALB/c WT mice (90%, H-2K^d+^; Fig. 6 E). We immunized these mice with NP-CGG in alum, treated them with tamoxifen at day 35, rechallenged them with NP-CGG on day 58, and analyzed them 21 d later on day 79. We found that deletion of BAFFR led to a large and significant reduction in the numbers of H-2K^b+^ IgG1^+^ MBCs and IgM^+^ and IgG1^+^ PCs in the spleen and bone marrow, while the numbers of control (H-2K^d+^) BAFFR-expressing MBCs and PCs remained unchanged (Fig. 6 F and Fig. S5 D). Thus, MBCs have a cell-intrinsic requirement for BAFFR to mount an effective recall response.

### Survival of MBCs is partially IKK1 dependent and strongly IKK2 dependent

**Figure 7.**
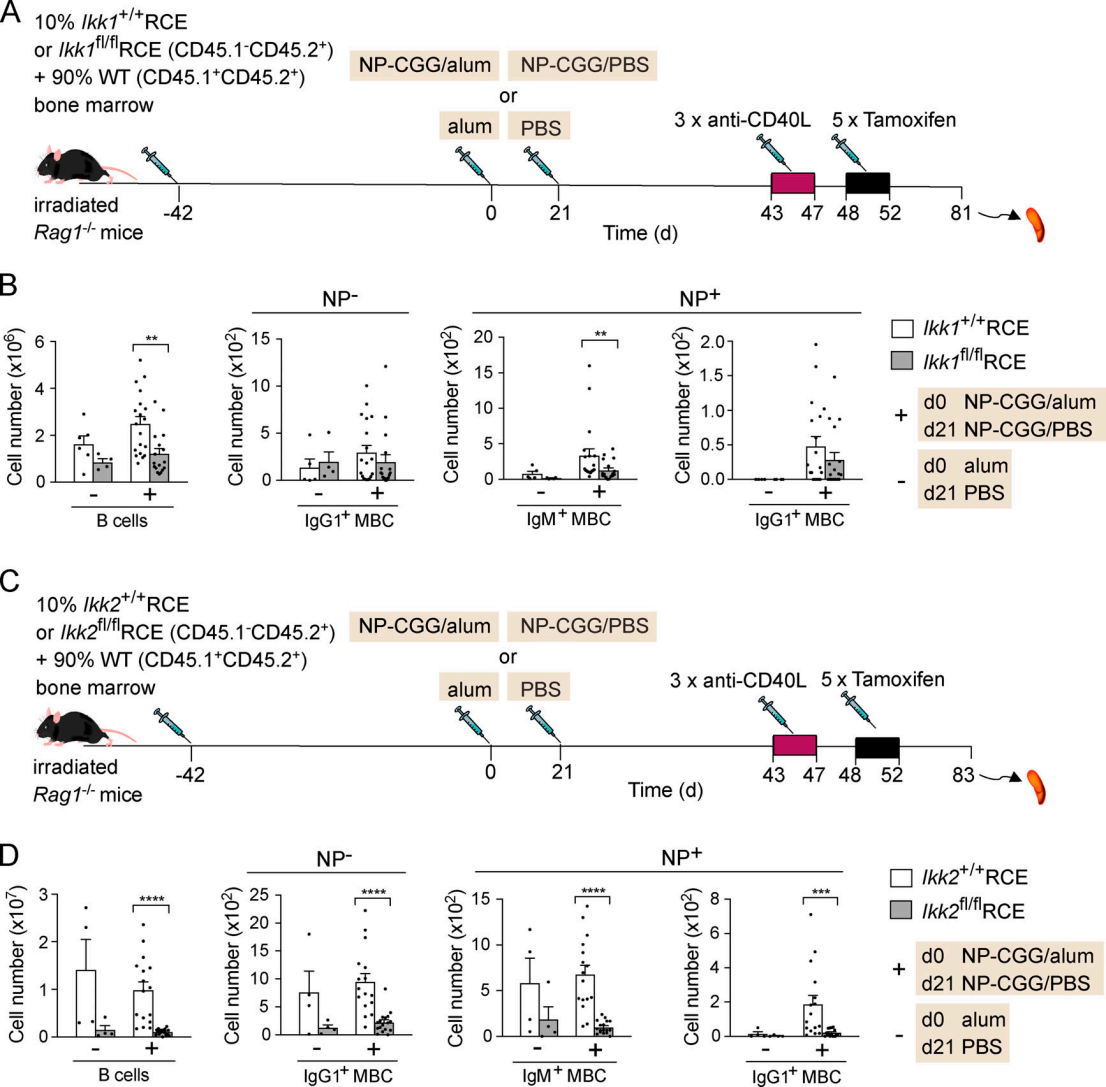
**Survival of MBCs is dependent on IKK2.** **(A)** Mixed bone marrow chimeras were generated by reconstituting irradiated *Rag1*^−/−^ mice with 10% bone marrow from *Ikk1*^+/+^RCE or *Ikk1*^fl/fl^RCE (CD45.1^−^CD45.2^+^) and 90% WT bone marrow (CD45.1^+^CD45.2^+^) for 42 d. Mice were immunized with NP-CGG in alum or alum alone on day 0, boosted with NP-CGG in PBS or PBS alone on day 21, and injected with anti-CD40L on days 43, 45, and 47, followed by five daily tamoxifen injections. **(B)** Mean (±SEM) numbers of total CD45.1^−^CD45.2^+^ B cells (CD138^−^B220^+^), NP^−^ IgG1^+^ MBCs, and NP^+^ IgM^+^ and IgG1^+^ MBCs (CD138^−^B220^+^Fas^−^PD-L2^+^) in spleens of mice immunized with NP-CGG in alum (+; *n* = 19 for *Ikk1*^+/+^RCE and *n* = 17 for *Ikk1*^fl/fl^RCE) or alum alone (−; *n* = 6 for *Ikk1*^+/+^RCE and *n* = 4 for *Ikk1*^fl/fl^RCE) were gated as shown in Fig. 4 B. **(C)** Mixed bone marrow chimeras were generated by reconstituting irradiated *Rag1*^−/−^ mice with 10% bone marrow from *Ikk2*^+/+^RCE or *Ikk2*^fl/fl^RCE (CD45.1^−^CD45.2^+^) and 90% WT (CD45.1^+^CD45.2^+^) bone marrow for 42 d. Mice were immunized with NP-CGG in alum or alum alone on day 0, boosted with NP-CGG in PBS or PBS alone on day 21, and injected with anti-CD40L on days 43, 45, and 47, followed by five daily tamoxifen injections. **(D)** Mean (±SEM) numbers of total CD45.1^−^CD45.2^+^ B cells, NP^−^ IgG1^+^ MBCs, and NP^+^ IgM^+^ and IgG1^+^ MBCs in spleens of mice immunized with NP-CGG in alum (+; *n* = 16 for *Ikk1*^+/+^RCE and *n* = 17 for *Ikk1*^fl/fl^RCE) or alum alone (−; *n* = 4) were gated as shown in Fig. 4 B. Each dot represents one mouse. Data pooled from three (B) and two (D) independent experiments. Mann-Whitney test was used for statistical analysis. **, 0.001 < P < 0.01; ***, 0.0001 < P < 0.001; ****, P < 0.0001.

IKK1 and IKK2 are kinases that transduce BAFFR signals to the noncanonical and canonical NF-κB signaling pathways, respectively, and may contribute to the pro-survival function of the receptor (Schweighoffer and Tybulewicz, 2018). To evaluate whether either kinase plays a role in MBC survival, we again made use of a genetic ablation strategy. We crossed *Ikk1*^fl/fl^ mice in which exons 5–9 of the *Chuk* gene are floxed (Gareus et al., 2007) with mice carrying the RCE allele, to allow tamoxifen-induced deletion of IKK1. To investigate a cell-intrinsic requirement for IKK1 in MBC survival, we generated radiation chimeras with a mixture of bone marrow from *Ikk1*^+/+^RCE or *Ikk1*^fl/fl^RCE (10%) mice and congenically marked WT bone marrow (90%). These chimeric mice were immunized with NP-CGG in alum or with alum alone, treated with anti-CD40L, and injected with tamoxifen to induce the deletion of *Ikk1* (Fig. 7 A). Loss of IKK1 resulted in an ∼50% decrease in numbers of mature B cells as well as NP^+^ IgM^+^ MBCs (Fig. 7 B). In contrast, the numbers of IgG1^+^ MBCs were partially reduced, but the decrease did not reach statistical significance. Thus, IKK1 is partially required for the maintenance of IgM^+^ MBCs but is less important for IgG1^+^ MBCs.

Finally, to investigate if IKK2 is required for MBC survival, we crossed *Ikk2*^fl/fl^ mice in which exon 3 of the *Ikbkb* gene is floxed (Park et al., 2002) to mice carrying the RCE allele and used these to generate *Ikk2*^+/+^RCE and *Ikk2*^fl/fl^RCE mixed bone marrow chimeras (Fig. 7 C). Chimeric mice were immunized with NP-CGG in alum and then treated with anti-CD40L and tamoxifen to induce deletion of *Ikk2*. Compared with *Ikk2*^+/+^RCE mice, loss of IKK2 resulted in a very large reduction of mature B cells as well as NP^+^ IgM^+^ and IgG1^+^ MBCs and NP^−^ IgG1^+^ MBCs in *Ikk2*^fl/fl^RCE mice (Fig. 7 D). Taken together, these results indicate that survival of IgM^+^ MBCs is partially dependent on IKK1, whereas both IgM^+^ and IgG1^+^ MBCs are strongly dependent on IKK2 for their maintenance.

## Discussion

We have established a genetic ablation system that allows study of the role of specific genes and their protein products in MBC maintenance. Importantly, by first generating a pool of MBCs specific to a known antigen, then blocking further MBC generation, and finally inducibly deleting a gene of choice, we were able to assess the role of the gene in MBC maintenance separately from any potential role in MBC development. Previously, only two proteins were known to be required for MBC maintenance—SYK and PLCγ2 (Ackermann et al., 2015; Hikida et al., 2009). In this study, we identified critical roles for five further proteins in this process—BCR, CD79A, BAFF, BAFFR, and IKK2.

Given that the SYK kinase and its target PLCγ2 are required for MBC survival (Ackermann et al., 2015; Hikida et al., 2009) and that both proteins transduce signals from the BCR, we speculated that BCR signaling might be required for MBC maintenance. Based on inducible deletion of the BCR or of the CD79A ITAM, our results demonstrate a critical cell-intrinsic requirement for BCR signaling in MBC survival. Since antigen is not required for MBC survival (Anderson et al., 2006; Hannum et al., 2000; Maruyama et al., 2000), we conclude that MBC survival requires antigen-independent BCR signaling, similar to the finding in naive B cells (Kraus et al., 2004). BCR ablation on naive B cells results in induction of cell death, suggesting that the loss of MBCs following deletion of the BCR may also be caused by apoptosis (Lam et al., 1997).

Analysis of MBCs with a deletion of the CD79A ITAM showed that both IgM^+^ and IgG1^+^ MBCs require BCR signaling for survival. However, for IgG1^+^ MBCs, dependency on the CD79A ITAM was only seen in competitive mixed chimeras but not in the absence of competition. This result demonstrates that switched MBCs can survive in part using CD79A-independent signals but that this is not sufficient when the cells are faced with competition from WT B cells and MBCs. Interestingly, the CD79A ITAM is also not required for the survival of naive B cells that had been engineered to express IgG1 in place of IgM, implying that IgG1 can signal independently of the CD79A ITAM (Waisman et al., 2007). In contrast to IgM, whose three–amino acid cytoplasmic domain has no known signaling capacity, the 28–amino acid cytoplasmic domain of IgG1 contains a highly conserved tyrosine residue that is phosphorylated when the IgG1 BCR is engaged by antigen (Engels et al., 2009). Mutation of this Ig tail tyrosine (ITT) results in a slow decline in numbers of IgG1^+^ MBCs over a period of several months, indicating that the ITT transduces survival signals from IgG1 (Lutz et al., 2015). Taken together, we speculate that the survival of IgM^+^ MBCs is dependent on BCR signals transduced via the CD79A ITAM, whereas IgG1^+^ MBCs may use either the CD79A ITAM or the ITT of IgG1. While the requirement for the CD79A ITAM for IgM^+^ and IgG1^+^ MBC survival is cell intrinsic, loss of the CD79A ITAM in IgG1^+^ MBCs makes the cells less able to compete with WT IgG1^+^ MBCs for cell-extrinsic factors, such as BAFF. These may be more limiting in the presence of a large excess of WT naive B cells and MBCs.

In contrast to a previous study that reported that murine MBCs do not express BAFFR (Benson et al., 2008), we find that both IgM^+^ and IgG1^+^ MBCs express BAFFR mRNA and have surface levels of the receptor similar to those seen on naive FO B cells. This difference may be due to use of a better anti-BAFFR detection reagent in our study or to improved sensitivity of flow cytometers. We note that a recent study has shown BAFFR on the surface of human MBCs (Smulski et al., 2017). Interestingly, both splenic and bone marrow MBCs have detectable levels of TACI on the surface compared with naive B cells, which do not.

Importantly, we show that BAFF and BAFFR are required for the maintenance of both IgM^+^ and IgG1^+^ MBCs in the spleen, bone marrow, and lung, generated either in response to a model antigen or to viral infection. Furthermore, we show that loss of MBCs following blockade of BAFF or deletion of BAFFR results in impaired secondary immune responses, demonstrating a requirement for BAFF and BAFFR in the maintenance of humoral immunological memory. Several previous studies had reported that BAFF was not required for MBC maintenance or for humoral recall responses (Benson et al., 2008; Rauch et al., 2009; Scholz et al., 2008), although one of these studies reported a partial dependence of splenic IgM^+^ MBCs on BAFF (Scholz et al., 2008). This difference may be due to the more stringent flow cytometric definition of MBCs in our studies, where we used PD-L2 expression and, in most cases, antigen specificity to positively identify MBCs. In addition, we investigated the recall response up to 3 wk after rechallenge, at which point the observed impairment in anti-BAFF–treated mice was greater than that seen at the 5–7 d time points used in previous studies (Benson et al., 2008; Scholz et al., 2008).

Interestingly, we find that in addition to the spleen, the bone marrow also serves as a critical survival niche for MBCs in mice. Our flow cytometric analysis showed that MBCs have TACI on the surface, and various cell types in the bone marrow express APRIL, a BAFF-family molecule that binds to TACI but not BAFFR, so the survival of bone marrow MBCs may also be supported in part by APRIL. Since PC survival in the bone marrow requires APRIL (Tangye, 2011), it will be interesting to investigate if MBCs use the same bone marrow survival niche as PCs. In support of such a possibility, a recent study showed that, similar to PCs, bone marrow MBCs bind to VCAM1^+^ stromal cells (Chang et al., 2018; Riedel et al., 2020; Tokoyoda et al., 2004; Zehentmeier et al., 2014).

BAFF plays an important role in the pathogenesis of various autoimmune diseases including systemic lupus erythematosus, and the anti-BAFF antibody belimumab has been approved as a treatment for systemic lupus erythematosus (Vincent et al., 2014). Analysis of blood from patients treated with belimumab showed a decrease in numbers of naive B cells but a transient increase in MBCs (Jacobi et al., 2010; Stohl et al., 2012). However, tissue-resident MBCs that make up the majority of this B cell population were not evaluated in these patients. It is therefore possible that the transient increase in numbers of MBCs in patient blood may have been caused by release from tissues, where their survival niche was destroyed by anti-BAFF treatment. However, in our mouse studies, we did not see such an increase in MBCs in the blood, potentially reflecting differences between mice and humans in the response of MBCs to BAFF blockade. Further work is needed to understand the relationship between circulating and tissue-resident MBCs.

We found that the loss of IKK1 results in a partial loss of naive B cells and IgM^+^ MBCs. This result appears to contradict a previous report showing that a B cell–specific inducible deletion of IKK1 does not affect mature B cell maintenance (Jellusova et al., 2013). This difference can be explained by a large excess of competing WT B cells in our studies, creating a more stringent test of survival. In contrast, Jellusova et al. (2013) deleted IKK1 in all B cells and had no competing WT B cells. Indeed, we have also seen that loss of IKK1 does not affect B cell numbers in a noncompetitive environment (data not shown). We note that loss of IKK1 has no significant effect on survival of IgG1^+^ MBCs even in the presence of competition, suggesting that there may be cell-intrinsic differences in the use of the noncanonical NF-κB signaling pathway in IgG1^+^ MBCs compared with IgM^+^ MBCs and naive B cells. It is possible that signaling from IgG1 through the ITT to GRB2 and Bruton's tyrosine kinase may compensate for loss of IKK1 (Engels et al., 2014).

IKK2 has been reported to be crucial for development of B cells (Pasparakis et al., 2002), but its requirement for B cell maintenance has never been tested. Here, we report for the first time that inducible IKK2 deletion leads to a strong reduction of total B cells as well as IgM^+^ and IgG1^+^ MBCs, suggesting a fundamental role for IKK2 in mature B cell and MBC survival. BAFFR activates the canonical NF-κB pathway via IKK2 (Rickert et al., 2011; Shinners et al., 2007). Furthermore, constitutively active IKK2 allows B cell survival in the absence of BAFFR (Sasaki et al., 2006). In view of these reports, taken together with our results, we propose that BAFFR transduces signals via IKK2 to NF-κB that are required for mature B cell and MBC survival. However, we cannot exclude the possibility that IKK2 may be transducing survival signals from other receptors.

Our results show that MBCs require signals through both BCR and BAFFR for survival, similar to the requirements of naive FO B cells. This raises the question of whether MBCs and FO B cells compete for the same survival signals. The number of new FO B cells arriving every day in the spleen is substantially higher than the total pool of splenic MBCs (Allman et al., 1993). If FO B cells and MBCs were to compete directly for the same survival factors, MBCs would be rapidly displaced. One possibility is that MBCs respond better to BAFF than naive B cells do, although we were unable to detect such a difference using an in vitro survival assay. We found that *Adam10* and *Adam17* are expressed at lower levels on MBCs than on naive cells, which may in turn result in lower rates of BAFFR shedding from the surface of MBCs, thereby prolonging BAFFR signaling. We also note that there are differences in the expression of Bcl-family proteins between MBCs and naive B cells, which may contribute to differences in life span. For example, IgM^+^ and IgG1^+^ MBCs express fivefold to eightfold more *Mcl1*, which codes for an anti-apoptotic Bcl-family protein (Table S1). Alternatively, MBC survival may also be regulated by other receptors such as LIFR or ADORA2A (Luckey et al., 2006; Tomayko et al., 2008). Further work is needed to investigate these possibilities.

In summary, we have shown that MBC survival is dependent on the BCR and its signaling subunit CD79A, on BAFF and BAFFR, and on the IKK2 kinase.

## Materials and methods

### Mice

Mice used in this study with the following alleles have been described before: loxP-flanked (floxed) NP^+^ VDJ region introduced into the IgH gene (*Igh*^tm4Cgn^, *Igh*^B1-8f^; Lam et al., 1997), NP^+^ VDJ region introduced into the IgH gene (*Igh*^tm2Cgn^, *Igh*^B1-8i^; Sonoda et al., 1997), *Cd79a* with a floxed exon encoding the ITAM domain (*Cd79a*^tm5Cgn^, *Cd79a*^C1f^; Kraus et al., 2004), floxed *Baffr* (*Tnfrsf13c*^tm1Mass^, *Baffr*^fl^; Sasaki et al., 2004), floxed *Ikk1* (*Chuk*^tm1Mpa^, *Ikk1*^fl^; Gareus et al., 2007), floxed *Ikk2* (*Ikbkb*^tm2Mka^, *Ikk2*^fl^; Park et al., 2002), tamoxifen-inducible CreER^T2^ in the ROSA26 locus (*Gt(ROSA)26Sor*^tm1(cre/ERT2)Thl^, RCE; de Luca et al., 2005), tamoxifen-inducible CreER^T2^ in the *Cd79a* gene (*Cd79a*^tm3(cre/ERT2)Reth^, mb1CreERT2, *Cd79a*^CE^; Hobeika et al., 2015), Blimp1GFP reporter (*Prdm1*^tm1Nutt^, Blimp1GFP; Kallies et al., 2004), RAG1-deficient (*Rag1*^tm1Mom^, *Rag1*^−/−^; Mombaerts et al., 1992), RAG2-deficient (*Rag2*^tm1Fwa^, *Rag2*^−/−^; Shinkai et al., 1992), and γ_c_-deficient (*Il2rg*^tm1Wjl^, *Il2rg*^−/−^; Cao et al., 1995). All mouse strains with mutations as well as WT C57BL/6J (B6), BALB/cJ (BALB/c), and B6 × B6.SJL (CD45.2^+^CD45.1^+^) mice were bred at the Francis Crick Institute. All mutant mouse strains were maintained on a B6 genetic background and intercrossed to generate required allelic combinations. All animal work was approved by the Francis Crick Institute’s Animal Welfare and Ethical Review Body and performed under a Project License granted by the UK Home Office.

### Bone marrow chimeras and adoptive transfer

To generate bone marrow chimeras, single-cell suspensions from tibiae and femurs of donor mice (*Cd79a*^wt/CE^ or *Cd79a*^C1f/CE^, *Baffr*^+/+^RCE or *Baffr*^fl/fl^RCE, *IKK1*^+/+^RCE or *IKK1*^fl/fl^RCE, and *IKK2*^+/+^RCE or *IKK2*^fl/fl^RCE) were depleted of erythrocytes using ACK lysis buffer (155 mM NH_4_Cl, 10 mM KHCO_3_, 100 μM EDTA) as previously described (Schweighoffer et al., 2013). At least 10^6^ cells were injected i.v. into *Rag1*^−/−^ recipient mice, which had been irradiated with 5 Gy using a ^137^Cs source ≤24 h before injection. For mixed chimeras, 10% bone marrow cells from mice carrying floxed alleles on a B6 background (CD45.2^+^CD45.1^−^) were mixed with 90% B6 × B6.SJL WT (CD45.2^+^CD45.1^+^) bone marrow before injection into *Rag1*^−/−^ recipient mice irradiated with 5 Gy. Alternatively, 10% bone marrow cells from mice carrying floxed alleles on a B6 background (H-2K^b+^) were mixed with 90% BALB/c (H-2K^d+^) bone marrow before injection into *Rag2*^−/−^*Il2rg*^−/−^ mice irradiated with 5 Gy. Mice were given water supplemented with 0.02% enrofloxacin (Baytril; Bayer Healthcare) for 4 wk after transplantation and used for experiments 7–10 wk after reconstitution. For adoptive transfer experiments, 10^7^ splenocytes from *Igh*^B1-8f/B1-8f^, *Igh*^B1-8f/B1-8f^RCE, *Igh*^B1-8f/+^RCE, or *Igh*^B1-8i/+^RCE mice (CD45.2^+^CD45.1^−^) were injected i.v. into WT (B6 × B6.SJL, CD45.2^+^CD45.1^+^) recipient mice that had been immunized with 50 µg CGG (Biosearch Technologies) precipitated in alum (Imject Alum; Thermo Fisher Scientific) 21 d earlier.

### Immunizations and influenza infection

MBCs were generated in a number of different ways. First, mice were immunized i.p. with PBS containing CGG or 50 µg NP conjugated at a ratio of 20–27 to CGG (NP_20-27_–CGG; Biosearch Technologies) that had been precipitated with alum or were immunized with alum precipitate alone. For recall responses, mice were injected i.p. with 50 µg NP_20-27_–CGG in PBS or PBS alone. To generate MBCs in response to influenza, B6 mice were infected i.n. with 30 µl PBS containing 5 × 10^3^ TCID_50_ (tissue culture infective dose 50%) of the influenza strain X31 or with PBS alone. Mice were monitored for weight loss and clinical symptoms for 10 d and were euthanized if they lost >20% of their starting weight.

### Administration of tamoxifen and antibodies

To block ongoing GC responses, immunized mice were injected i.p. on 3 alternate d with 300 µg anti-CD40L (MR-1; BioXCell). To induce Cre recombinase activity, 2 mg of tamoxifen (20 mg/ml in corn oil; Sigma Aldrich) was administered i.p. daily for 5 d. BAFF depletion in mice was achieved by two i.p. injections of 100 µg anti-BAFF (10F4; GSK) or, as a control, with Armenian hamster IgG (BioXCell).

### Flow cytometry

Blood was collected in heparin-coated tubes, and lymphocytes were isolated using Histopaque-1077 (Sigma Aldrich). Single-cell suspensions from spleen and bone marrow were depleted of erythrocytes using ACK lysis buffer as previously described (Schweighoffer et al., 2013), before staining cells with a mixture of antibodies in PBS containing the live/dead marker Zombie Aqua (BioLegend). Antibodies against the following antigens were used (indicating antigen-fluorophore [clone]): B220-BV605 (RA3-6B2), B220-FITC, B220-PerCP/Cy5.5, B220-BV785, CD3–APC/Cy7 (145-2c11), CD11b-bio (M1/70), CD23-PacificBlue (B3B4), CD23–PerCP/Cy5.5, H-2K^d^-FITC (SF1-1.1), IgG2b–PE/Cy7 (RMG2b-1), and IgG2b-FITC from BioLegend; BAFFR-FITC (7H22-E16), CD4-PacificBlue (RM4-5), CD8–PerCP/Cy5.5 (53–6.7), CD73–PE/Cy7 (eBioTY-11.8), CD93-APC (AA4.1), CD45.1–PerCP/Cy5.5 (A20), CD45.2-APC-eF780 (104), and NK1.1–PE/Cy7 (PK136) from eBioscience; CD19–PerCP/Cy5.5 (1D3), CD138-PE (281–2), CD138-BUV737, BAFFR-BV711, Fas-PE-CF594 (Jo2), Fas-BV421, H-2K^b^–PerCP/Cy5.5 (AF6-88.5), IgG1-BUV395 (X56), IgA-FITC (C10-1), IgG1-APC, IgA-bio, PD-L2-BV421 (Ty25), and TACI-AF647 (8F10) from BD Biosciences. Goat–anti-mouse IgG2c-FITC (STAR135F) was purchased from Bio-Rad, anti–IgG3-bio (SB76b) and anti–IgG3-FITC from Southern Biotech, goat–anti-mouse IgM Fab-FITC and -AF647 from Jackson ImmunoResearch, and NP-PE from Biosearch Technologies. To conjugate PE to X31 HA (Sino Biological), the LYNX Rapid Ab Conjugation Kit for PE (AbD Serotec) was used. Data were analyzed using FlowJo v10.5 (TreeStar).

### ELISA

To measure antibody or BAFF levels in the serum, blood was withdrawn from the tail vein. NP^+^ IgM and IgG1 serum titers were measured by ELISA using Maxisorp plates (Nunc) coated with 5 µg/ml NP_20_-BSA (Biosearch Technologies) as previously described (Hartweger et al., 2014). BAFF serum titers were detected using the Mouse BAFF ELISA Kit (Tnfsf13b; Abcam).

### ELISPOT

Single-cell suspensions from spleen and bone marrow of mice 21 d after rechallenge were depleted of erythrocytes and seeded into NP_20_-BSA–coated nitrocellulose 96-well filtration plates (Millipore) as previously described (Ackermann et al., 2015). Spots, which represent NP^+^ IgM or IgG1 secretion by individual PCs, were counted using an ImmunoSpot Analyzer (Cellular Technology Limited).

### In vitro survival assay

Splenic B cells were purified by depletion of CD43^+^ cells and incubated for 0, 3, and 5 d with or without recombinant human BAFF (PeproTech). Total numbers of live MBCs were determined by flow cytometry.

### RNAseq

FO B cells were sorted from spleens of naive B6 female mice. GC B cells were isolated by flow sorting from female B6 mice immunized 10 d earlier with NP-CGG in alum. To isolate MBCs, B6 female mice were immunized with NP-CGG in alum, and splenic IgM^+^ and IgG1^+^ MBCs were purified by flow sorting 35 d after immunization. To isolate PBs and PCs, Blimp1GFP mice were immunized with NP-CGG in alum, and splenic PBs and PCs and bone marrow PCs were purified by flow sorting 35 d after immunization. Three replicates of each cell population (1,000 cells each) were sorted directly into TRIzol (Thermo Fisher Scientific) using the Aria FUSION, InFlux v7 (BD Biosciences) or MoFlo XDP (Beckman Coulter) sorter. RNA was purified using the RNeasy Plus Micro kit (QIAgen) according to the manufacturer’s instructions. Un-stranded, non–ribosomal RNA, non-polyA^+^ selected libraries were prepared using the SMARTer Ultra Low Input RNA kit for Sequencing v3 (Clontech Laboratories). The libraries were sequenced on the Illumina HiSeq 2000 platform as 50-bp paired-end runs, generating 10.4–82 million reads per sample. Cutadapt v1.9.1 (Martin, 2011) was used to trim adapter sequences from reads with the following options: -a AGATCGGAAGAGC –A AGATCGGAAGAGC -e 0.1 –minimum length 30 –q 20,20. Gene-level expected counts were calculated with RSEM v1.2.31 (Li and Dewey, 2011), using STAR v2.5.1b (Dobin et al., 2013) to align reads against the GRCm38 genome assembly with Ensembl release 86 transcript annotations. Count data were rounded to integers and normalized for differences in sample library size using the standard median ratio method in DESeq2 (Anders and Huber, 2010). Table S1 shows mean normalized counts for all genes in the seven sorted B cell populations, as well as normalized counts for Bcl-family genes and for genes encoding proteins involved in BAFFR signaling. All RNAseq data have been deposited in Gene Expression Omnibus with accession no. GSE133971.

### Statistical analysis

Nonparametric two-tailed Mann-Whitney *U* test was performed for all statistical analyses using Prism (GraphPad). Statistically significant differences are indicated in the figures: *, 0.01 < P < 0.05; **, 0.001 < P < 0.01; ***, 0.0001 < P < 0.001; ****, P < 0.0001.

### Online supplemental material

Fig. S1 shows analysis of BCR deletion in *Igh*^B1-8f/B1-8f^RCE mice at 3, 6, and 10 d following the start of tamoxifen treatment. Fig. S2 shows the analysis of B cell and MBC numbers in WT cells in mixed chimeras that also contained CD79A- or BAFFR-deficient B cells. Fig. S3 shows the flow cytometric gating strategies used to sort different B cell populations for RNAseq as well as for flow cytometric analysis of BAFFR and TACI expression. Fig. S4 shows the flow cytometric gating strategies used for the analysis of MBCs in mice that had been immunized with NP-CGG or infected with influenza and then treated with anti-BAFF, as well as analysis of BAFF-dependent survival of B cells and MBCs in vitro. Fig. S5 shows the ELISPOT assay used to determine numbers of antibody-secreting cells in the study of the effect of anti-BAFF on recall response and shows the flow cytometric gating strategies used to analyze H-2K^b+^ and H2-K^d+^ MBCs in the study of the recall response in mixed bone marrow chimeras in which BAFFR had been deleted in some of the B cells. Table S1 shows mean normalized counts for all genes in the seven sorted B cell populations, as well as normalized counts for Bcl-family genes and for genes encoding proteins involved in BAFFR signaling.

## Supplementary Material

Table S1 shows mean normalized counts for all genes in the seven sorted B cell populations, as well as normalized counts for Bcl-family genes and for genes encoding proteins involved in BAFFR signaling.
